# New α-galactosidase-inhibiting aminohydroxycyclopentanes†‡

**DOI:** 10.1039/d1ra02507d

**Published:** 2021-04-29

**Authors:** Patrick Weber, Roland Fischer, Seyed A. Nasseri, Arnold E. Stütz, Martin Thonhofer, Stephen G. Withers, Andreas Wolfsgruber, Tanja M. Wrodnigg

**Affiliations:** Glycogroup, Institute of Chemistry and Technology of Biobased Systems, Graz University of Technology Stremayrgasse 9 A-8010 Graz Austria patrick.weber@tugraz.at +43 316 873 32078; Institute of Inorganic Chemistry, Graz University of Technology Stremayrgasse 9 A-8010 Graz Austria; Chemistry Department, University of British Columbia 2036 Main Mall Vancouver BC V6T 1Z1 Canada

## Abstract

A set of cyclopentanoid α-galactosidase ligands was prepared from a partially protected ω-eno-aldose *via* a reliable (2 + 3)-cycloaddition protocol with slightly modified conditions. The obtained *N*-benzylisoxazolidine ring was selectively opened and the configuration of the hydroxymethylgroup was inverted. Consecutive deprotection provided an aminocyclopentane, which was *N*-alkylated to furnish a set of potential α-galactosidase inhibitors. Their glycosidase inhibitory activities were screened with a panel of standard glycosidases of biological significance.

## Abstract 2

A concise and robust synthesis of new cyclopentanoid competitive inhibitors of α-galactosidases related to Fabry's disease and other α-galactosidase related disorders.

## Graphical abstract

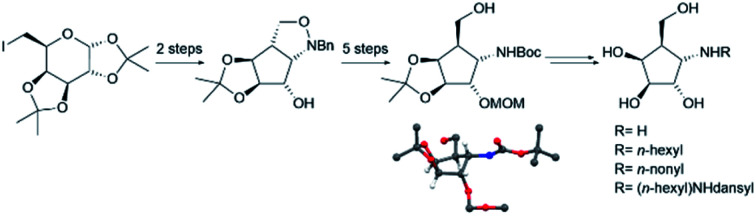

## 1. Introduction

Glycoside hydrolases are a class of abundant and essential enzymes for carbohydrate catabolism, controlled trimming of oligosaccharide motifs in glycoproteins, as well as the release of stored polysaccharides in plants and animals. The Carbohydrate-Active enZYmes database^1,2^ (CAZY) currently features well over one hundred and sixty sequence-associated families of glycosidases.

α-d-Galactosides are, in a quite diverse context, an important class of oligosaccharides and carbohydrate conjugates. Their corresponding hydrolases, α-d-galactosidases, are found in glycoside hydrolase (GH) families 4, 27 (containing the Fabry disease related human lysosomal enzyme), 36, 57, 97 and 110 as well as in GH109. GH 4 and GH109 enzymes follow a mechanism requiring NAD^+^ and Mn^2+^ that starts with the oxidation at C-3 followed by β-elimination of the aglycone, with subsequent water addition and reduction,^3^ while the enzymes of the other five families follow standard Koshland retaining mechanisms.^4^

α-d-Galactosidases have been employed or modulated in several quite diverse applications. For example, α-galactosidases are frequently employed in combination with proteases to remove oligosaccharides from food to improve their nutritional availability and quality.^5,6^ Improving the activity of lysosomal α-galactosidase through chaperoning is a new approach to the management of Fabry's disease, a hereditary lysosomal disorder resulting from mutations in the GLA gene that result in catalytically compromised mutants of this essential enzyme.^7–11^ In a completely different context, α-galactosidases that selectively and efficiently remove the immunodominant α-1,3-galactosyl epitope (Galili antigen) which hampers xenotransplantation from otherwise suitable mammalian sources, have been a focus of organ and tissue transplant medicine.^12–14^ Similarly, for the purpose of generating red blood cells for readily available universal donor blood, enzymatic conversion of B type red blood cells into type O, by removal of α-1,3-bound galactosides from the cell surface, has been a field of intense and sustained research.^15–19^

Specific inhibitors of α-galactosidases have been developed as chaperones for treatment of the hereditary lysosomal disorder, Fabry's disease. A prime example is 1,5-dideoxy-1,5-imino-d-galactitol (DGJ, 1), which enhances the residual activity of various α-galactosidase mutants, ameliorating Fabry related symptoms and providing improved quality of life for afflicted patients. α-Galactosidase inhibitors may also help the discovery, purification and characterization of enzymes for managed degradation of pathologically active α-galactosides.

Amongst competitive α-galactosidase inhibitors, compound 1 is the current bench mark (IC_50_ 1.6 nM and 0.24 μM with α-galactosidases from green coffee beans (gcb, GH 27) and *E. coli*, (GH 36) respectively).^20^ Homolog 2 inhibits gcb galactosidase with IC_50_ 0.06 μM and *E. coli* enzyme with IC_50_ 32 μM.^21^ Contrasting this, structurally related 4-*epi*-isofagomine 3 exhibits β-galactosidase activity practically exclusively (IC_50_ 200 μM, gcb).^22^ Conversely, *N*-alkyl substituted analogues of the corresponding 5-fluoro derivative, such as 4, are sub-micromolar (*K*_i_ 0.50 μM) inhibitors of gcb α-galactosidase.^23^ Pyranoid carbasugar 4-*epi*-validamine (5) is only a weak inhibitor with IC_50_ 500 μM (gcb) and 890 μM (*E. coli*), respectively.^24^ Interestingly, closely related bicyclic analogue 6 is much more potent with *K*_i_ 0.54 μM and IC_50_ 80 μM with these enzymes.^25^

Turning to furanoid structures, 1,4-dideoxy-1,4-imino-d-lyxitol (7) was found to be an inhibitor of gbc α-galactosidase (*K*_i_ 0.13 μM).^26^ Its C-6-homolog, 2,5-dideoxy-2,5-imino-d-altritol (8), exhibited an IC_50_ of 0.78 μM with α-galactosidase from green coffee beans without any noteworthy inhibition of a panel of other glycosidases.^27^ Other researchers reported an IC_50_ of 5.2 μM for compound 8 with the same enzyme.^28^ These authors also noticed that *meso*-2,5-dideoxy-2,5-iminogalactitol (10) was an even better inhibitor (IC_50_ 0.19 μM) of gcb α-galactosidase while d-*galacto* configured aminocyclopentane 12 inhibits it with *K*_i_ 0.43 μM.^29^ In contrast to that, aminocyclopentane 12 does not show any inhibitory activity against bovine liver α-galactosidase.^30^ Furthermore, a study by Zheng and co-workers^31^ revealed that non-carbohydrate-based structures such as lansoprazole (IC_50_ 6.4 μM) may also serve as gcb α-galactosidase inhibitors.

**Fig. 1:**
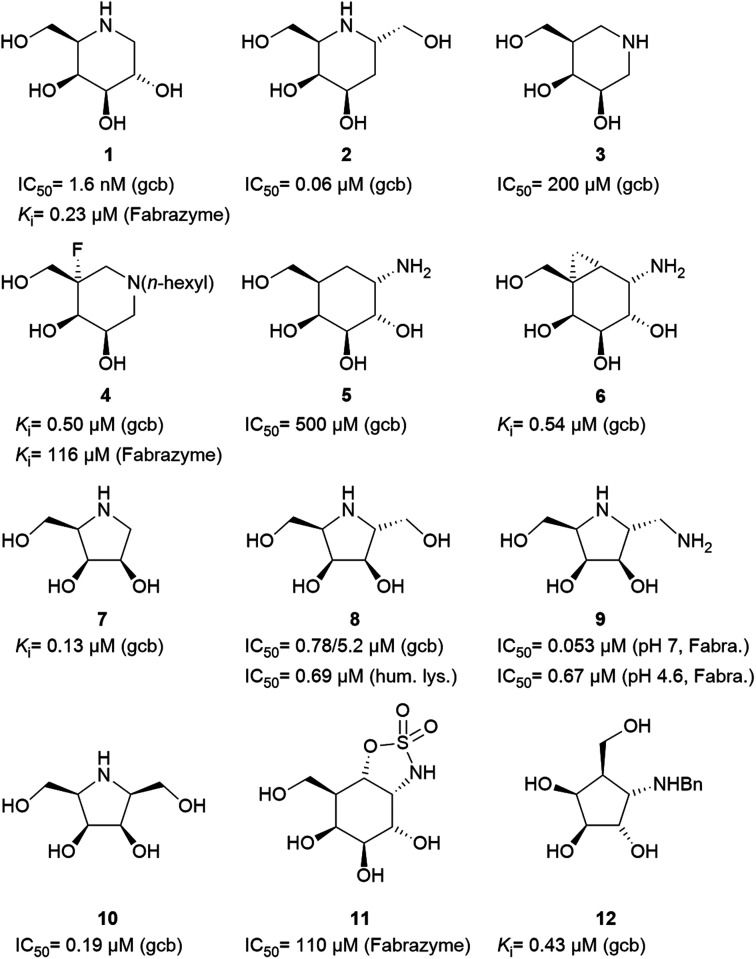
Carbohydrate-related α-galactosidase inhibitors and inhibitory activities.

Human lysosomal α-galactosidase (Fabrazyme®, GH 27) is strongly inhibited by compounds 1 (*K*_i_ 0.23 μM, Fabrazyme®),^32^4 (*K*_i_ 116 μM, Fabrazyme®),^23^8 (IC_50_ 0.69 μM, human lysosomal),^27^ and moderately by 11 (*K*_i_ 110 μM, Fabrazyme®).^32^ Compound 9, the 1-aminodeoxy derivative of 8, exhibits IC_50_ 0.053 μM (pH 7) and IC_50_ 0.67 μM (pH 4.6) with Fabrazyme®^33^ (Fig. 1). These data demonstrate that only very few small glycomimetics known thus far exhibit noteworthy activity as well as selectivity for α-galactosidases.

We have recently been interested in hydroxymethyl-branched di- and trihydroxycyclopentylamines such as compound 12, which may be regarded as resulting from ring contraction by formal homolytic extraction of the sugar ring oxygen and bond formation between C-1 and C-5. The new carbacyclic scaffold maintains the stereochemical information of the corresponding parent free sugar or glycosylamine albeit with the characteristic conformational features of five-membered rings. There are only four leading references in which such sugar analogues are shown as proven d-galactosidase inhibitors.^29,30,34,35^ As already outlined, there is only one *N*-alkylated hydroxymethyl-trihydroxycyclopentylamine 12 reported in literature. Yet, this compounds shows sufficient inhibitory activity against α-galactosidase (0.43 μM, gbc) but also inhibitory activity against β-galactosidase (1.5 μM, bovine liver) as well as β-glucosidase (0.51 μM, almonds). Aim of this work, is the synthesis of new aminocyclopentanes, as powerful and selective GH27 α-galactosidase inhibitors. These structures were prepared, relying on Vasella's (2 + 3)-cycloaddition approach.^36^

## 2. Results and discussion

### 2.1. Synthesis

**Scheme 1:**
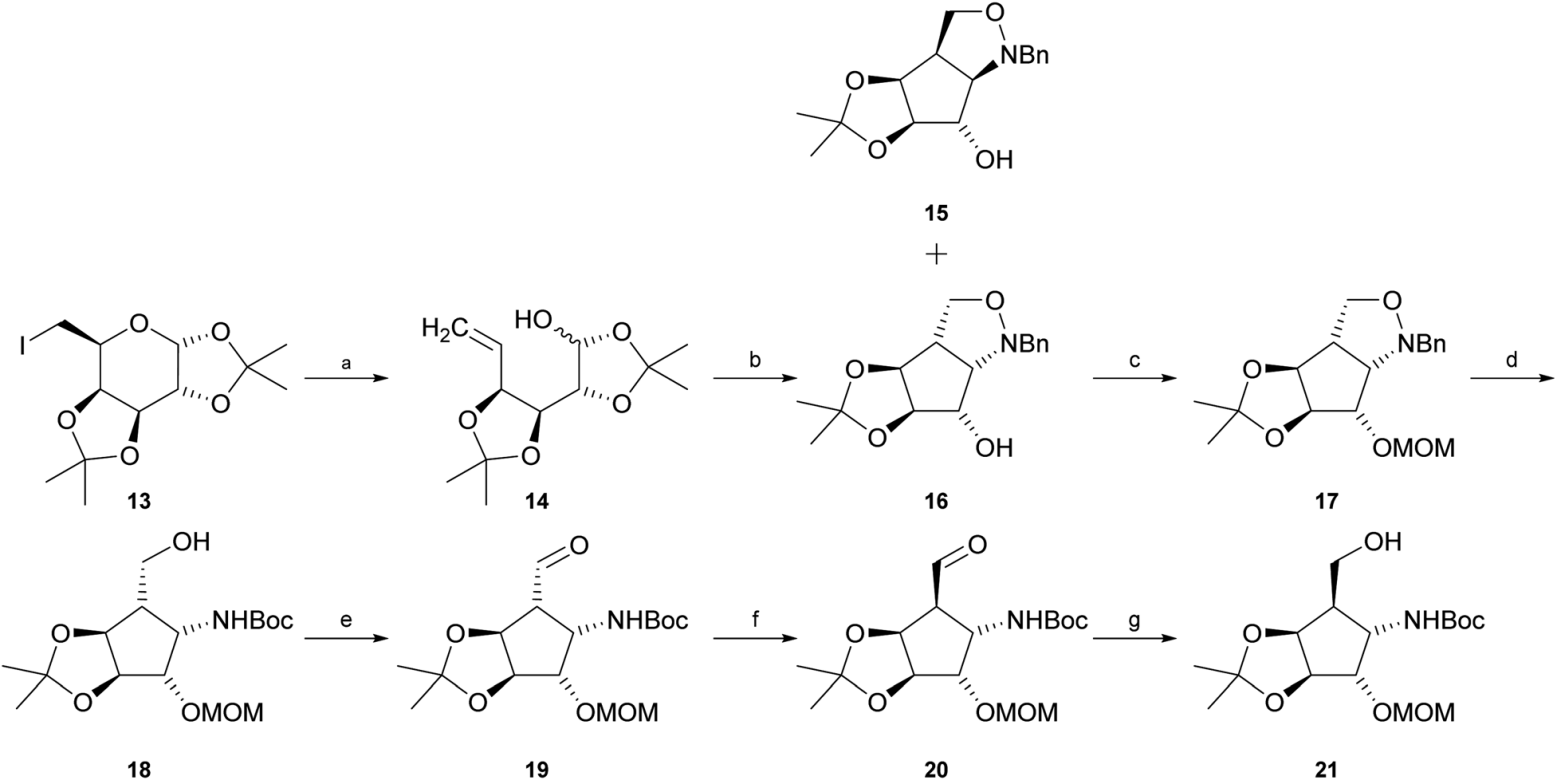
Synthesis of intermediate 21. (a) Zn, NH_4_Cl, MeOH; (b) BnNHOH·HCl, pyridine, CH_2_Cl_2_, 75% over two steps, 86% by conversion; (c) MOMCl, i-Pr_2_NEt, CH_2_Cl_2_, reflux, 91%; (d) H_2_, 20% Pd(OH)_2_/C, Boc_2_O, Na_2_CO_3_, MeOH, 90%; (e) Dess–Martin periodinane, CH_2_Cl_2_ or oxalyl chloride, DMSO, i-Pr_2_NEt, CH_2_Cl_2_, −60 °C; (f) pyridine or i-Pr_2_NEt, CH_2_Cl_2_; (g) DIBAL-H, CH_2_Cl_2_, 0 °C, 47% over three steps, 62% by conversion.

Intermediate 21 was synthesised starting with reductive elimination of known iodosugar 13 (ref. 37) with zinc under slightly acidic conditions to give hemiacetal 14. Its treatment with *N*-benzylhydroxylamine in CH_2_Cl_2_, provided a mixture of *syn*-product 15 and the desired known^38,39^*anti*-configured *N*-benzylisoxazolidine 16 (experimental details as well as NMR-data had not been reported for this compound) in a ratio of about 1 : 7 (15 : 16). The stereochemical outcome of this cyclisation is highly dependent on the solvent. Jäger and co-workers^39^ pointed out that if the reaction is carried out in a polar solvent such as methanol, the main product is compound 15. In contrast, usage of an unpolar solvent such as CH_2_Cl_2_ or CHCl_3_ mainly provides compound 16. Separation of compounds 15 and 16 was easily achieved by silica gel chromatography. Protection of the free hydroxyl function in compound 16 with chloromethyl methyl ether (MOMCl) in the presence of Hünig's base (i-Pr_2_NEt) yielded fully protected compound 17. “One-pot” hydrogenolytic removal of the *N*-benzyl group over Pd(OH)_2_/C (20%) in the presence of Boc_2_O (di-*tert*-butyl-dicarbonate) and Na_2_CO_3_ resulted in *N*-Boc-protected primary alcohol 18. Subsequent Swern or Dess–Martin oxidation furnished the corresponding aldehyde 19. Treatment of aldehyde 19 with pyridine or i-Pr_2_NEt in methanol allowed, *via* enolization, almost complete isomerisation to epimer 20. This reaction could easily be followed by thin-layer chromatography. The epimerization reaction was then quenched by addition of NaBH_4_ at 0 °C to obtain desired primary alcohol 21. Unexpectedly, the conditions of this attempted *in situ* reduction reaction favoured re-isomerization at C-5, now exclusively – *via* intermediate 19 – providing starting alcohol 18, possibly influenced by unfavourable boronate complexation of aldehyde 19 or by easier access of the reagent *trans* to the isopropylidene group and consequently higher reactivity of the “wrong” aldehyde epimer, 19, in the equilibrium. Improvement of experimental conditions by isomerisation of aldehyde 19 in CH_2_Cl_2_ in the presence of i-Pr_2_NEt to obtain desired epimer 20, and consecutive *in situ* reduction with diisobutylaluminium hydride (DIBAL-H) in CH_2_Cl_2_ resulted in a mixture of desired alcohol 21 and intermediate 18 in a ratio of about 2 : 1 (21 : 18). The diastereomers were separated chromatographically to obtain pure 21 (47%) and recovered starting material 18 (25%) (Scheme 1).

**Fig. 2:**
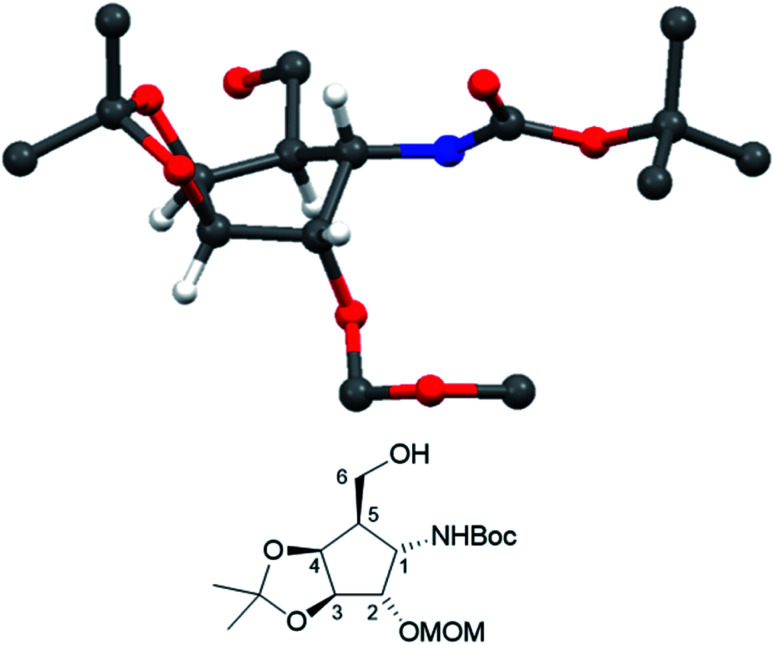
Crystal structure of compound 21. (CCDC: 2065318, for the sake of consistency, numbering follows the monosaccharide pattern starting with the amino substituted carbon (C-1) clockwise along the ring.)

Crystallization from cyclohexane/ethyl acetate afforded crystals of sufficient quality to verify the structural identity of diastereomer 21 by X-ray structure determination (Fig. 2, CCDC 2065318).

**Scheme 2:**
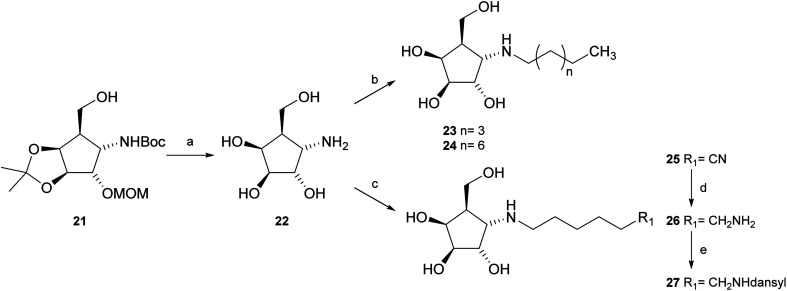
Synthesis of inhibitors 23, 24 and 27. (a) Trifluoroacetic acid, THF–MeOH–H_2_O (3 : 1 : 1), 60 °C, 75%; (b) R–CHO, AcOH, H_2_, 20% Pd(OH)_2_/C, MeOH, 57% (23), 64% (24); (c) Br(CH_2_)_5_CN, NaHCO_3_, DMF, 60 °C; (d) H_2_, RANEY®-Ni, MeOH, 49%, two steps; (e) dansyl chloride, Na_2_CO_3_, MeOH, 55%.

Deprotection of alcohol 21 with trifluoroacetic acid in THF–MeOH–H_2_O (3 : 1 : 1) resulted in known^30^ aminotetraol 22. Reductive amination with the respective aldehyde in the presence of Pd/C under an atmosphere of H_2_ gave chain extended inhibitors 23 and 24, respectively. Alkylation of amine 22, by treatment with ω-bromo hexanoic nitrile furnished compound 25, which was subsequently reduced in the presence of RANEY®-Ni under an atmosphere of H_2_ to obtain primary amine 26. Its conventional *N*-dansylation yielded inhibitor 27 (Scheme 2).

### 2.2. Inhibitory activities

Amines 22–24 and 27 were screened for inhibitory activities with a panel of standard glycosidases (Table 1). As can be seen the compounds act as inhibitors of both α- and β-galactosidases, though overall they perform better on α-galactosidases with inhibition constants down to the high nanomolar/low micromolar range, as discussed in more detail below.

**Table 1:** *K*_i_-values [μM] of compounds 1, 8, 22–24, 27^a^

Enzyme (GH family)	Compounds					
	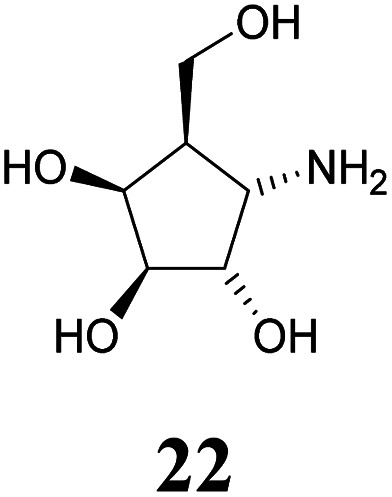	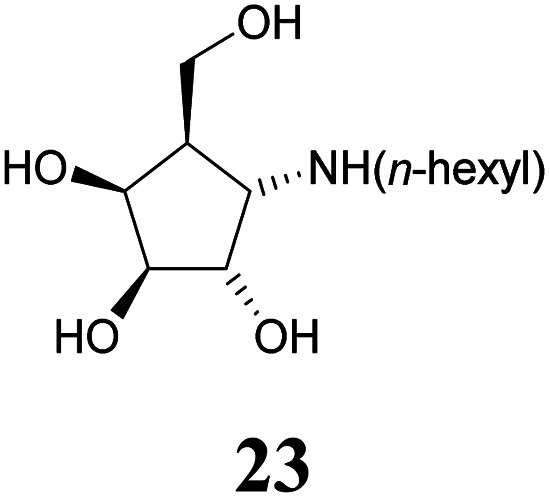	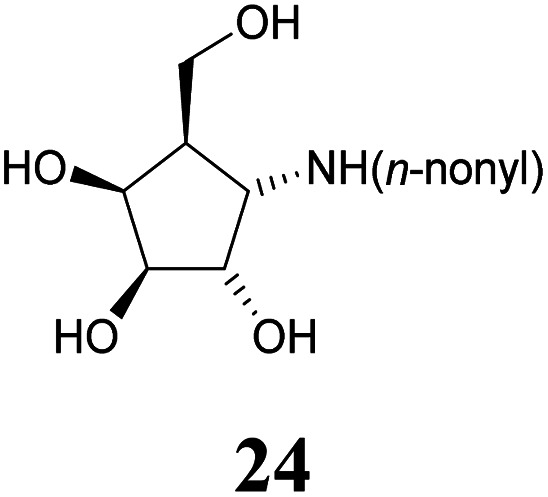	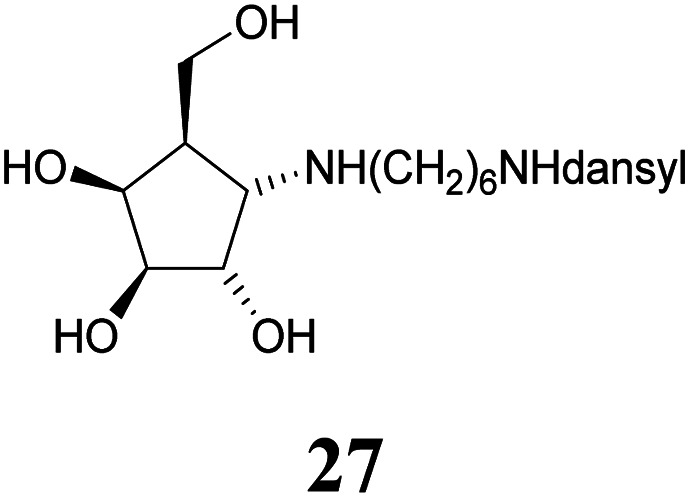	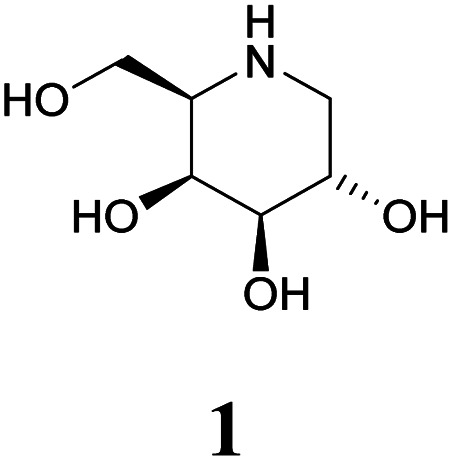	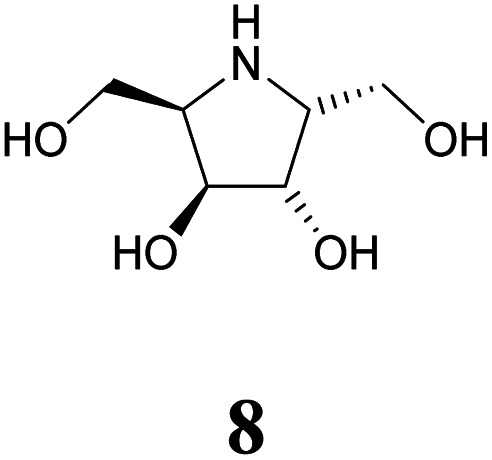
Abg (β-Glc/β-Gal) (GH 1)	0.014 ± 0.003	0.013 ± 0.003	0.0037 ± 0.0016	0.005 ± 0.001	12 (ref. 40)	n.d.
*E*. *coli* (β-Gal) (GH 2)	200 ± 90	120 ± 20	23 ± 7	7.8 ± 4.6	13 (ref. 40)	n.d.
Bovine liver (β-Gal) (GH 35)	1.4 ± 1.0 (3.3 (ref. 30))	0.093 ± 0.054	0.19 ± 0.07	0.18 ± 0.08	n.i.^41^	IC_50_: 190 (ref. 27)
Fabrazyme® (α-Gal) (GH 27)	9.5 ± 1.9	4.9 ± 0.9	0.52 ± 0.21	4.4 ± 1.1	0.23 (ref. 32)	IC_50_: 0.69 (ref. 27) (h. lys.)
gcb (α-Gal) (GH 27)	6.7 ± 0.9 (12 (ref. 30))	0.19 ± 0.05	0.25 ± 0.05	0.14 ± 0.04	0.0016 (ref. 40)	IC_50_: 0.78 (ref. 27)
*S. cer.* (α-Glc) (GH 13)	160 ± 60	130 ± 40	73 ± 17	16 ± 4	n.i.^41^ (yeast)	n.d.
GCase (β-Glc) (GH 30)	18 ± 3	40 ± 8	1.2 ± 0.5	0.63 ± 0.09	n.d.	n.d.

a Abg = β-glucosidase/β-galactosidase from *Agrobacterium* sp.; *E. coli* = lac *Z* β-galactosidase from *E. coli*; bovine liver = β-galactosidase from bovine liver; Fabrazyme® = commercial recombinant lysosomal α-galactosidase; gcb = α-galactosidase from green coffee beans *S. cer*. = α-glucosidase from *S. cerevisiae*; GCase = recombinant human lysosomal β-glucocerebrosidase. h. lys. = human lysosomal α-galactosidase. n.d. = not determined, n.i. = no inhibition, with *K*_i_ values estimated to be >1 mM.

**Fig. 3:**
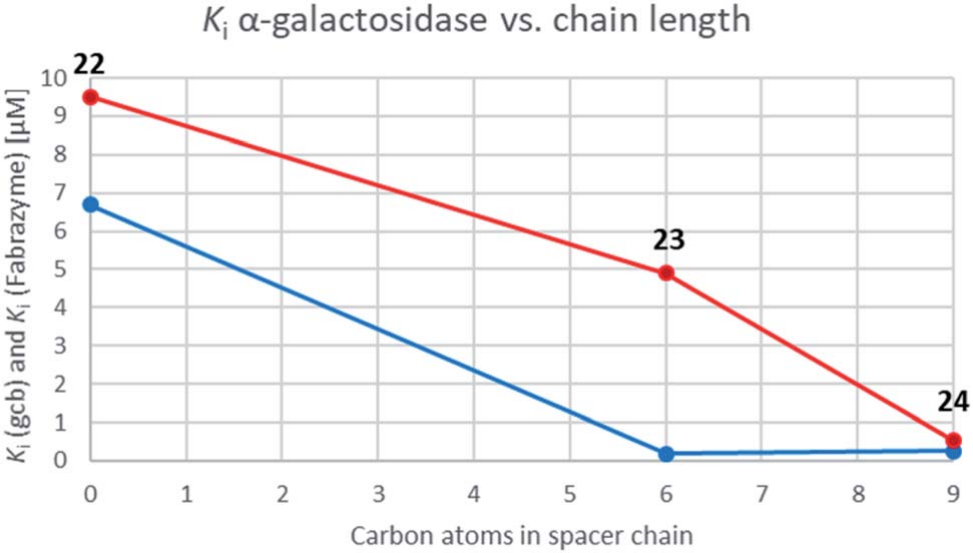
Graphs correlating inhibition constants of gcb α-galactosidase (blue) as well as Fabrazyme® (red) *vs.* spacer chain length.

While the parent molecule with a free amine proved to be a modest inhibitor with inhibition constants in the low to mid micromolar range, alkylation of this nitrogen improved inhibition in each case (with one exception in the case of GCase). Further extension of the alkyl chain generally improved inhibition yet more indeed for the β-glycosidases, all of which belong to the same, structurally related clan GHA, largely parallel increases in inhibition were seen with each addition of carbon atoms. This is consistent with common interactions with closely related structures.^42,43^ For the α-galactosidases, the alkylation also generally improved inhibition, but not as predictably as with the β-galactosidases, despite the fact that both, Fabrazyme® and gcb, belong to the same sequence-related family, GH27 (Fig. 3).

Optimal affinity for the human enzyme, Fabrazyme®, was obtained with the *n*-nonyl substituted derivative 24, with a *K*_i_ value comparable to that of galacto-isofagomine. This is the compound in current clinical use under the name Migalastat® or Galafold. The presence of the *n*-nonyl chain in 24 would likely improve its pharmacokinetic profile by keeping the compound in circulation longer, making this a candidate for testing as a chaperone. However, the fact that this compound is also a good inhibitor of other glycosidases tested here, means that its selectivity needs to be further improved.

## 3. Conclusions

Following a simple protocol previously outlined by Vasella's and Jäger's groups,^35,36^ a series of novel derivatives of 12, bearing chain extensions at the nitrogen at position C-1 have been synthesized. Inhibition constants for these compounds with a range of d-galactosidases, including human lysosomal α-galactosidase, were then measured. The results of these inhibitory studies revealed that free amine 22 is the worst inhibitor within this series and the activity increases with the introduction of an alkyl chain as seen with compounds 23, 24 as well as 27.

Furthermore, it is both unexpected and interesting that the α-configured compounds developed here are good inhibitors of the β-glycosidases in the first place, since these have the wrong anomeric configuration for these enzymes. Further work will elucidate the reason for this unexpected finding.

## 4. Experimental

### 4.1. General methods

Optical rotations were measured at 20 °C on a Perkin Elmer 341 polarimeter at a wavelength of 589 nm and a path length of 10 cm. [*α*]^20^_D_ values are given in 10^−1^ degree cm^2^ per g. NMR spectra were recorded on a Bruker Ultrashield spectrometer at 300.36 (^1^H) and 75.53 MHz (^13^C). CDCl_3_ was employed for protected compounds and CD_3_OD or D_2_O for unprotected inhibitors. Carbon and hydrogen numbering in NMR spectra was conducted in analogy to carbohydrate nomenclature and clockwise, starting with the amino bearing carbon as C-1 (Fig. 2). Chemical shifts are listed in delta employing residual, non-deuterated solvent as the internal standard. Signals were assigned unambiguously by COSY, HSQC as well as APT analysis. Coupling constants ‘*J*’ for protecting groups, alkyl chains as well as the dansyl group (spacer arms) were found in the expected range and are not listed. For intermediate 21, the structure was confirmed by XRD structural analysis: suitable single crystals of compounds were immersed in silicone oil, mounted using a glass fiber and frozen in the cold nitrogen stream (100 K). X-Ray diffraction data were collected at low temperature on a Bruker Kappa APEX II diffractometer using Mo K_α_ radiation (*l* = 0.71073 Å) generated by an INCOATEC micro-focus source. The data reduction and absorption correction was performed with the Bruker SMART and Bruker SADABS, respectively. The structures were solved with SHELXT^44^ by direct methods and refined with SHELXL^45^ by least-square minimization against *F*^2^ using first isotropic and later anisotropic thermal parameters for all non-hydrogen atoms. Hydrogen atoms were added to the structure models on calculated positions using the riding model. The space group assignments and structural solutions were evaluated using PLATON.^46,47^ MALDI-TOF Mass Spectrometry was performed on a Micromass TofSpec 2E Time-of-Flight Mass Spectrometer. Analytical thin-layer chromatography (TLC) was performed on precoated aluminium plates silica gel 60 F254 (E. Merck 5554) and detected with UV light (254 nm). For staining, a solution of vanillin (9 g) in a mixture of H_2_O (950 mL)/ethanol (750 mL)/H_2_SO_4_ (120 mL) or ceric ammonium molybdate (100 g ammonium molybdate/8 g ceric sulfate in 10% H_2_SO_4_ (1 L)) were employed followed by heating on a hotplate. For column chromatography, silica gel 60 (230–400 mesh, E. Merck 9385) or silica gel 60 (Acros Organics, AC 24036) were used. Reaction monitoring was done by TLC.

### 4.2. Kinetic studies

Kinetic studies were performed at room temperature in an appropriate buffer for each enzyme (specific conditions can be found below). All reactions were performed in half-area 96-well-plates (Corning) and monitored with a Synergy H1 plate reader (BioTek). In each experiment, the appropriate concentration of the enzyme was incubated with different concentrations of the inhibitors for 2–5 minutes before initiating the reaction by the addition of the glycoside substrate. The initial rate was then measured by monitoring the increase in, either absorbance at 405 nm (where the aglycone is either *p*-nitrophenol (pNP) or 2,4-dinitrophenol (dNP)) or fluorescence at 365/455 nm (where the aglycone is 4-methylumbelliferone (MU)) for up to five minutes. *K*_i_ determinations were performed using two different substrate concentrations. For each substrate concentration, a range of three to six inhibitor concentrations was used. Dixon plots (1/*v vs.* [*I*]) were constructed to validate the use of competitive inhibition model and to assess the fit of the data. The data were then fit to a competitive inhibition model using non-linear regression analysis with Grafit 7.0.0. (Erithacus Software).

Specific assay conditions for each enzyme:

*Agrobacterium* sp. β-glucosidase:^48,49^ 50 mM sodium phosphate buffer (pH 7) substrate: Gal-β-pNP (*K*_m_ = 4.1 mM).

*E. coli* lac *z* β-galactosidase: 50 mM sodium phosphate containing 1.0 mM MgCl_2_ (pH 7) substrate: Gal-β-pNP (*K*_m_ = 60 μM).

Bovine liver β-galactosidase: 50 mM sodium phosphate buffer (pH 7) substrate: Gal-β-pNP (*K*_m_ = 0.65 mM).

Green coffee bean α-galactosidase: 50 mM sodium phosphate buffer (pH 7) substrate: Gal-α-MU, *K*_m_ = 250 μM.

Fabrazyme® (acid α-galactosidase): 20 mM sodium citrate, 50 mM sodium phosphate, 1.0 mM tetrasodium EDTA, 0.25% v/v Triton X-100® and 0.25% w/v taurocholic acid buffer (pH 5.5) substrate Gal-α-dNP (*K*_m_ = 0.65 mM).

*S. cerevisiae* α-glucosidase: 50 mM sodium phosphate buffer (pH 7). Substrate: Glc-α-pNP, *K*_m_ = 0.75 mM.

GCase (β-glucocerebrosidase): 20 mM sodium citrate, 50 mM sodium phosphate, 1.0 mM tetrasodium EDTA, 0.25% v/v Triton X-100® and 0.25% w/v taurocholic acid buffer (pH 7) susbtrate: Glc-β-dNP (*K*_m_ = 2.7 mM).

#### (3a*S*,3b*S*,6a*S*,7*S*,7a*S*)-1-Benzyl-5,5-dimethylhexahydro-1*H*-[1,3]dioxolo[4′,5′:3,4]cyclopenta[1,2-*c*]isoxazol-7-ol (16)

To a solution of known iodosugar^37^13 (5.38 g, 14.5 mmol) in methanol (100 mL), zinc (11.4 g, 174 mmol) and NH_4_Cl (2.33 g, 43.6 mmol) were added. After completed conversion (15 min, cyclohexane–EtOAc 3 : 1) the solids were removed by filtration and the filtrate was concentrated under reduced pressure. The residue containing crude 14 was taken up in CH_2_Cl_2_ (100 mL) and this suspension was treated with pyridine (3.45 mL, 43.6 mmol) and *N*-benzylhydroxylamine hydrochloride (2.78 g, 17.4 mmol). After consumption of the starting material (TLC, 3 h, cyclohexane–EtOAc 2 : 1) the suspension was consecutively washed with saturated aqueous NaHCO_3_ solution and brine. The combined organic layers were dried (Na_2_SO_4_) and the solvents were removed under reduced pressure to obtain a yellow syrup. Purification on silica gel (cyclohexane–EtOAc 6 : 1 to 1 : 1) provided desired *anti*-configurated *N*-benzylisoxazolidine 16 (3.19 g, 10.9 mmol, 75% over two steps, 86% by conversion, *R*_f_ = 0.6). The melting point as well as the optical rotation [*α*]^20^_D_ were in accordance with reported^38^ data. ^1^H-NMR (300 MHz, CDCl_3_) *δ* = 7.40–7.24 (m, 5H, aromatics), 4.51 (bs, 2H, *J*_2,3_ < 1 Hz, *J*_3,4_ < 1 Hz, *J*_4,5_ < 1 Hz, H-3, H-4), 4.24 (dd, 1H, *J*_5,6a_ = *J*_6a,6b_ 8.6 Hz, H-6a), 4.14 (d, 1H, N–CH̲_2_–Ph), 3.98 (dd, 1H, *J*_1,2_ 5.9 Hz, H-2), 3.94 (dd, 1H, *J*_1,5_ 6.0 Hz, H-1), 3.86 (d, 1H, N–CH̲_2_-Ph), 3.68 (dd, 1H, *J*_5,6b_ 5.3 Hz, H-6b), 3.66 (bs, 1H, OH), 3.22 (dddd, 1H, H-5), 1.45, 1.31 (2 s, 3H each, C(CH̲_3_)_2_). ^13^C-NMR (75.5 MHz, CDCl_3_): *δ* = 136.6 (*ipso*), 129.1, 128.8, 128.0 (aromatics), 111.2 (C̲(CH_3_)_2_), 87.5, 84.0 (C-3, C-4), 75.6 (C-2), 71.3 (C-1), 70.2 (C-6), 61.4 (N–C̲H_2_–Ph), 54.1 (C-5), 27.6, 25.2 (C(C̲H_3_)_2_). MS (MALDI): calculated for [C_16_H_21_NO_4_H]: *m*/*z* 292.1549 [M + H]^+^; found [M + H]^+^ 292.1547.

The already reported^35^ corresponding *syn*-configurated *N*-benzylisoxazolidine 15 (438 mg, 1.50 mmol, 10.3%) was obtained as colourless solid.

#### (3a*S*,3b*S*,6a*S*,7*S*,7a*S*)-1-Benzyl-7-(methoxymethoxy)-5,5-dimethylhexahydro-1*H*-[1,3]dioxolo[4′,5′:3,4]cyclopenta[1,2-*c*]isoxazole (17)

A solution of isoxazolidine 16 (3.79 g, 13.0 mmol) in CH_2_Cl_2_ (50 mL) was treated with i-Pr_2_NEt (11.1 mL, 65.0 mmol) and MOMCl (2.47 mL, 32.5 mmol) and the solution was stirred under reflux. After consumption of the starting material (24 h, cyclohexane–EtOAc 2 : 1), methanol (20 mL) was added and the solution was stirred for 20 min. The reaction mixture was allowed to reach ambient temperature and was consecutively washed with HCl (2 M) and saturated aqueous NaHCO_3_. The combined organic layers were dried (Na_2_SO_4_), filtered and concentrated under reduced pressure. The residue was purified on silica gel (cyclohexane–EtOAc 5 : 1) to provide compound 17 as a colourless oil (3.96 g, 11.8 mmol, 91%). [*α*]^20^_D_: −15.7 (*c* = 1.1, CHCl_3_); ^1^H-NMR (300 MHz, CDCl_3_) *δ* = 7.46–7.24 (m, 5H, aromatics), 4.78 (dd, 1H, *J*_2,3_ 5.1 Hz, *J*_3,4_ < 1 Hz, H-3), 4.71 (dd, 2H, O–CH̲_2_–O–CH_3_), 4.46 (dd, 1H, *J*_4,5_ 5.9 Hz, H-4), 4.25 (dd, 1H, *J*_1,2_ 5.5 Hz, H-2), 4.21 (dd, 1H, *J*_5,6a_ = *J*_6a,6b_ 8.6 Hz, H-6a), 4.05 (d, 1H, N–CH̲_2_-Ph), 3.73 (dd, 1H, *J*_5,6b_ 4.5 Hz, H-6b), 3.58 (dd, 1H, *J*_1,5_ 7.0 Hz, H-1), 3.40 (s, 3H, O–CH_2_–O–CH̲_3_), 3.16 (dddd, 1H, H-5), 1.53, 1.34 (2s, 3H each, C(CH̲_3_)_2_). ^13^C-NMR (75.5 MHz, CDCl_3_): *δ* = 137.7 (ipso), 129.2, 128.4, 127.4 (aromatics), 112.1 (C̲(CH_3_)_2_), 95.7 (O–C̲H_2_–O–CH_3_), 84.8 (C-3), 83.7 (C-4), 82.0 (C-2), 71.0 (C-1), 70.3 (C-6), 62.1 (N–C̲H_2_–Ph), 55.6 (O–CH_2_–O–C̲H_3_), 50.8 (C-5), 27.9, 25.6 (C(C̲H_3_)_2_). MS (MALDI): calculated for [C_16_H_21_NO_4_H]: *m*/*z* 336.1811 [M + H]^+^; found [M + H]^+^ 336.1818.

#### *tert*-Butyl ((3a*S*,4*S*,5*S*,6*S*,6a*S*)-4-(hydroxymethyl)-6-(methoxymethoxy)-2,2-dimethyltetrahydro-4*H*-cyclopenta[*d*][1,3]dioxol-5-yl)carbamate or 1-(*tert*-butyloxycarbonyl)amino-3,4-*O*-isopropylidene-2-*O*-methoxymethyl-β-l-*altro*-cyclopentane (18)

Isoxazolidine 17 (3.96 g, 11.8 mmol) was dissolved in methanol (50 mL) and was subsequently treated with Na_2_CO_3_ (2.50 g, 23.6 mmol), Boc_2_O (3.87 g, 17.7 mmol) and 20% Pd(OH)_2_/C (0.8 g). The suspension was stirred under H_2_ atmosphere at ambient pressure. After completed conversion (8 h, cyclohexane–EtOAc 1 : 1) the solids were removed by filtration. The solvent was removed under reduced pressure and the residue was chromatographed (cyclohexane–EtOAc 3 : 1) to furnish alcohol 18 (3.71 g, 10.7 mmol, 90%) as a colourless oil. [*α*]^20^_D_: −2.0 (*c* = 0.87, CHCl_3_); ^1^H-NMR (300 MHz, CDCl_3_) *δ* = 5.02 (dd, 1H, *J*_1,NH_ 7.1 Hz, NH), 4.73 (dd, 2H, O–CH̲_2_–O–CH_3_), 4.59 (dd, 1H, *J*_3,4_ < 1 Hz, *J*_4,5_ 6.9 Hz, H-4), 4.46 (dd, 1H, *J*_2,3_ 5.8 Hz, H-3), 4.42 (dd, 1H, *J*_1,2_ < 1 Hz, *J*_1,5_ 7.0 Hz, H-1), 4.05 (dd, 1H, H-2), 3.72–3.59 (m, 2H, H-6), 3.39 (s, 3H, O–CH_2_–O–CH̲_3_), 3.09 (bs, 1H, OH), 2.53 (m, 1H, H-5), 1.44 (s, 12H, C(CH̲_3_)_2_, NH–CO–O–C(CH̲_3_)_3_), 1.27 (s, 3H, C(CH̲_3_)_2_). ^13^C-NMR (75.5 MHz, CDCl_3_): *δ* = 156.1 (NH–C̲O–O–(C(CH_3_)_3_)), 111.1 (C̲(CH_3_)_2_), 96.7 (O–C̲H_2_–O–CH_3_), 82.8 (C-3), 82.3 (C-2), 80.6 (C-4), 80.1 (NH–CO–O–(C̲(CH_3_)_3_)), 59.9 (C-6), 56.4 (O–CH_2_–O–C̲H_3_), 52.9 (C-1), 49.4 (C-5), 28.5 (NH–CO–O–(C(C̲H_3_)_3_)), 26.6, 24.1 (C(C̲H_3_)_2_). MS (MALDI): calculated for [C_16_H_29_NO_7_H]: *m*/*z* 348.2022 [M + H]^+^; found [M + H]^+^ 348.2021.

#### *tert*-Butyl ((3a*S*,4*R*,5*S*,6*S*,6a*S*)-4-(hydroxymethyl)-6-(methoxymethoxy)-2,2-dimethyltetrahydro-4*H*-cyclopenta[*d*][1,3]dioxol-5-yl)carbamate or 1-(*tert*-butyloxycarbonyl)amino-3,4-*O*-isopropylidene-2-*O*-methoxymethyl-α-d-*galacto*-cyclopentane (21)

A solution of oxalyl chloride (1.04 mL, 12.1 mmol) in CH_2_Cl_2_ (40 mL) was treated with DMSO (1.03 mL, 14.5 mmol) at −60 °C. After 15 min, a solution of alcohol 18 (1.68 g, 4.84 mmol) in CH_2_Cl_2_ (5 mL) was added and the reaction was stirred for 15 min when i-Pr_2_NEt (4.93 mL, 29.0 mmol) was added. After completed oxidation (30 min, cyclohexane–EtOAc 1 : 1), the reaction mixture was consecutively washed with HCl (2 M) and saturated aqueous NaHCO_3_ solution. The collected organic layers were combined, dried (Na_2_SO_4_), filtered and concentrated under reduced pressure. The remaining syrup was quickly passed through a pad of silica gel (CH_2_Cl_2_ to EtOAc) and the solvents were removed under reduced pressure. The mixture of desired aldehyde 20 (cyclohexane–EtOAc 1 : 1, *R*_f_ = 0.6) and precursor 19 (cyclohexane–EtOAc 1 : 1, *R*_f_ = 0.7) was dissolved in CH_2_Cl_2_ (20 mL) and treated with i-Pr_2_NEt (1.64 mL, 9.67 mmol) at 0 °C. The reaction mixture was stirred until only the desired diastereomer 20 was observed (1 h, cyclohexane–EtOAc 1 : 1). Then DIBAL-H (9.67 mL, 1 M in CH_2_Cl_2_) was dropwise added to the solution. After completed reduction (30 min, cyclohexane–EtOAc 1 : 1), 20 mL of saturated aqueous potassium sodium tartrate solution were added. The mixture was vigorously stirred overnight and was consecutively washed with HCl (2 M) and saturated aqueous NaHCO_3_ solution, the combined organic layers were dried (Na_2_SO_4_), filtered and concentrated under reduced pressure. The residue was purified on silica gel (cyclohexane–EtOAc 4 : 1) to give alcohol 21 (785 mg, 2.26 mmol, *R*_f_ = 0.32, 47%, 62% by conversion) as a colourless oil. Epimer 18 (418 mg, 1.20 mmol, *R*_f_ = 0.40, 25%) was recovered. Crystallisation from EtOAc–cyclohexane gave colourless crystals of 21 with sufficient quality for XRD. Mp = 113–115 °C; [*α*]^20^_D_: −56.0 (*c* = 0.77, CHCl_3_); ^1^H-NMR (300 MHz, CDCl_3_) *δ* = 5.02 (bs, 1H, NH), 4.71 (dd, 1H, *J*_3,4_ = *J*_4,5_ 6.0 Hz, H-4), 4.70 (dd, 2H, O–CH̲_2_–O–CH_3_), 4.46 (dd, 1H, *J*_2,3_ < 1 Hz, H-3), 4.19–4.04 (m, 1H, H-1), 3.95 (dd, 1H, *J*_1,2_ 4.7 Hz, H-2), 3.91–3.80 (m, 2H, H-6), 3.38 (s, 3H, O–CH_2_–O–CH̲_3_), 2.33 (bs, 1H, OH), 2.12–2.01 (m, 1H, H-5), 1.44 (s, 12H, C(CH̲_3_)_2_, NH–CO–O–C(CH̲_3_)_3_), 1.28 (s, 3H, C(CH̲_3_)_2_). ^13^C-NMR (75.5 MHz, CDCl_3_): *δ* = 156.1 (NH–C̲O–O–(C(CH_3_)_3_)), 110.8 (C̲(CH_3_)_2_), 96.5 (O–C̲H_2_–O–CH_3_), 81.3, 81.2 (C-2, C-3), 79.9 (NH–CO–O–(C̲(CH_3_)_3_)), 79.0 (C-4), 60.6 (C-6), 56.0 (O–CH_2_–O–C̲H_3_), 52.4 (C-2), 48.2 (C-5), 28.5 (NH–CO–O–(C(C̲H_3_)_3_)), 26.0, 23.9 (C(C̲H_3_)_2_). MS (MALDI): calculated for [C_16_H_21_NO_4_H]: *m*/*z* 348.2022 [M + H]^+^; found [M + H]^+^ 348.2022.

#### (1*S*,2*S*,3*S*,4*S*,5*R*)-4-Amino-5-(hydroxymethyl)cyclopentane-1,2,3-triol or 1-amino-α-d-*galacto*-cyclopentane (22)

A solution of protected compound 21 (692 mg, 1.99 mmol) was dissolved in THF–MeOH–H_2_O (3 : 1 : 1, 10 mL) and treated with 5 mL trifluoroacetic acid. The solution was heated to 60 °C until completed deprotection was observed (12 h, CHCl_3_–MeOH–NH_4_OH (25%) 4 : 4 : 1). The solvents were removed under reduced pressure. The remaining syrup was co-evaporated with toluene (3 times) and was purified by silica gel chromatography (CHCl_3_–MeOH–NH_4_OH (25%) 8 : 4 : 1) to provide polyol 22 (245 mg, 1.50 mmol, 75%) as colourless solid. The corresponding hydrochloride was obtained by addition of conc. HCl to a methanolic solution of amine 22 (10 mg) adjusting the pH value to 1. Removal of the solvents under reduced pressure yielded known^30^22·HCl. The optical rotation was in accordance to the already reported.^30^ free base: ^1^H-NMR (300 MHz, D_2_O) *δ* = 4.15 (dd, 1H, *J*_3,4_ = *J*_4,5_ 4.6 Hz, H-4), 3.99 (dd, 1H, *J*_1,2_ = *J*_2,3_ 7.4 Hz, H-2), 3.91 (dd, 1H, H-3), 3.83–3.70 (m, 2H, H-6), 3.05 (dd, 1H, *J*_1,5_ 8.1 Hz, H-1), 2.00–1.89 (m, 1H, H-5). ^13^C NMR (75.5 MHz, D_2_O) *δ* = 78.0 (C-3), 74.8 (C-2), 70.1 (C-4), 59.9 (C-6), 51.4 (C-1), 49.4 (C-5). Hydrochloride: ^1^H-NMR (300 MHz, D_2_O) *δ* = 4.28 (dd, 1H, *J*_1,2_ = *J*_2,3_ 7.8 Hz, H-2), 4.23 (dd, 1H, *J*_3,4_ = *J*_4,5_ 4.7 Hz, H-4), 4.02 (dd, 1H, H-3), 3.87 (dd, 1H, *J*_5,6a_ 7.3 Hz, *J*_6a,6b_ 11.0 Hz, H-6a), 3.77 (dd, 1H, *J*_5,6b_ 7.1 Hz, H-6b), 3.59 (dd, 1H, *J*_1,5_ 8.3 Hz, H-1), 2.34 (dddd, 1H, H-5). ^13^C NMR (75.5 MHz, D_2_O) *δ* = 77.9 (C-3), 72.3 (C-2), 70.1 (C-4), 59.5 (C-6), 51.9 (C-1), 45.9 (C-5). MS (MALDI): calculated for [C_6_H_13_NO_4_H]: *m*/*z* 164.0923 [M + H]^+^; found [M + H]^+^ 164.0922.

#### (1*S*,2*S*,3*S*,4*S*,5*R*)-4-(*n*-Hexylamino)-5-(hydroxymethyl)cyclopentane-1,2,3-triol or 1-(*n*-hexyl)amino-α-d-*galacto*-cyclopentane (23)

A solution of amine 22 (24.5 mg, 0.150 mmol) in methanol (2 mL) was stirred in the presence of AcOH (20 μL), *n*-hexanal (27.7 μL, 0.225 mmol) and 20% Pd(OH)_2_/C (50 mg) under an atmosphere of H_2_ at ambient pressure. The suspension was stirred until complete consumption of the starting material was overserved (24 h, CHCl_3_–MeOH 3 : 1 + 1 vol% NH_4_OH (25%)). The catalyst was filtered off and the solvents were removed under reduced pressure. The residue was quickly passed through a pad of silica gel (CHCl_3_–MeOH 8 : 1 + 1 vol% NH_4_OH (25%)) to obtain inhibitor 23 (21.3 mg, 86.1 μmol, 57%) as a colourless solid. [*α*]^20^_D_: −23.5 (*c* = 0.82, MeOH); ^1^H-NMR (300 MHz, CD_3_OD) *δ* = 4.25 (dd, 1H, *J*_3,4_ 4.4 Hz, *J*_4,5_ 6.8 Hz, H-4), 4.05 (dd, 1H, *J*_1,2_ 6.5 Hz, *J*_2,3_ 4.1 Hz, H-2), 3.88 (dd, 1H, H-3), 3.79 (ddd, 2H, *J*_5,6a_ = *J*_5,6b_ 5.4 Hz, *J*_6a,6b_ 12.2 Hz, H-6), 3.30 (dd, 1H, *J*_1,5_ 7.5 Hz, H-1), 2.82 (t, 2H, H-1′), 2.16–2.04 (m, 1H, H-5), 1.65–1.26 (m, 8H, H-2′, H-3′, H-4′, H-5′), 0.91 (t, 3H, H-6′). ^13^C NMR (75.5 MHz, CD_3_OD) *δ* = 79.7 (C-3), 74.1 (C-2), 72.2 (C-4), 61.8 (C-6), 61.7 (C-1), 48.7 (C-1′), 47.8 (C-5), 32.7, 29.1, 27.7, 23.6 (C-2′, C-3′, C-4′, C-5′), 48.7 (C-6′). MS (MALDI): calculated for [C_12_H_25_NO_4_H]: *m*/*z* 248.1862 [M + H]^+^; found [M + H]^+^ 248.1862.

#### (1*S*,2*S*,3*S*,4*S*,5*R*)-4-(*n*-Nonylamino)-5-(hydroxymethyl)cyclopentane-1,2,3-triol or 1-(*n*-nonyl)amino-α-d-*galacto*-cyclopentane (24)

A methanolic solution (2 mL) of amine 22 (23.8 mg, 0.146 mmol) was treated with AcOH (20 μL) and *n*-nonanal (37.6 μL, 0.219 mmol) and stirred in the presence of 20% Pd(OH)_2_/C (50 mg) under an atmosphere of H_2_ at ambient pressure. After completed reaction (24 h, CHCl_3_–MeOH 3 : 1 + 1 vol% NH_4_OH (25%)), the catalyst was removed by filtration and the filtrate was concentrated under reduced pressure. Silica gel filtration (CHCl_3_–MeOH 8 : 1 + 1 vol% NH_4_OH (25%)) of the residue provided inhibitor 24 (26.8 mg, 92.6 μmol, 64%) as a colourless oil. [*α*]^20^_D_: −22.7 (*c* = 1.1, MeOH); ^1^H-NMR (300 MHz, CD_3_OD) *δ* = 4.22 (dd, 1H, *J*_3,4_ 4.8 Hz, *J*_4,5_ 6.2 Hz, H-4), 4.00 (dd, 1H, *J*_1,2_ 6.2 Hz, *J*_2,3_ 4.4 Hz, H-2), 3.86 (dd, 1H, H-3), 3.84–3.72 (m, 2H, H-6), 3.12 (dd, 1H, *J*_1,5_ 7.6 Hz, H-1), 2.68 (t, 2H, H-1′), 2.05–1.91 (m, 1H, H-5), 1.62–1.19 (m, 14H, H-2′, H-3′, H-4′, H-5′, H-6′, H-7′, H-8′), 0.90 (t, 3H, H-9′). ^13^C NMR (75.5 MHz, CD_3_OD) *δ* = 79.8 (C-3), 74.6 (C-2), 72.2 (C-4), 62.1 (C-6), 61.6 (C-1), 49.2 (C-1′), 48.7 (C-5), 33.0, 30.6, 30.6, 30.4, 30.2, 28.3, 23.7 (C-2′, C-3′, C-4′, C-5′, C-6′, C-7′, C-8′), 14.4 (C-9′). MS (MALDI): calculated for [C_15_H_31_NO_4_H]: *m*/*z* 290.2331 [M + H]^+^; found [M + H]^+^ 290.2335.

#### (1*S*,2*S*,3*S*,4*S*,5*R*)-4-((6-Aminohexyl)amino)-5-(hydroxymethyl)cyclopentane-1,2,3-triol or 1-(6-aminohexyl)amino-α-d-*galacto*-cyclopentane (26)

To a solution of amine 22 (51.7 mg, 0.612 mmol) in DMF (2 mL), NaHCO_3_ (154 mg, 1.84 mmol) and 6-bromohexanenitrile (106 μL, 0.796 mmol) were added at 60 °C. The suspension was stirred until complete conversion of the starting material was observed (24 h, CHCl_3_–MeOH 3 : 1 + 1 vol% NH_4_OH (25%)) and thereafter the solvent was removed under reduced pressure. The residue was chromatographically purified (CHCl_3_–MeOH 14 : 1 + 1 vol% NH_4_OH (25%)) to obtain crude nitrile 25 as a colourless oil. ^1^H-NMR (300 MHz, CD_3_OD) *δ* = 4.21 (dd, 1H, *J*_3,4_ 4.6 Hz, *J*_4,5_ 6.4 Hz, H-4), 3.98 (dd, 1H, *J*_1,2_ 6.5 Hz, *J*_2,3_ 4.2 Hz, H-2), 3.86 (dd, 1H, H-3), 3.84–3.76 (m, 2H, H-6), 3.08 (dd, 1H, *J*_1,5_ 8.1 Hz, H-1), 2.72–2.61 (m, 2H, H-1′), 2.47 (t, 1H, H-5′), 2.00–1.89 (m, 1H, H-5), 1.75–1.42 (m, 6H, H-2′, H-3′, H-4′). ^13^C NMR (75.5 MHz, CD_3_OD) *δ* = 121.2 (C-6′), 79.9 (C-3), 74.8 (C-2), 72.3 (C-4), 62.2 (C-6), 61.6 (C-1), 49.1 (C-5), 49.0 (C-1′), 29.8 (C-2′), 27.4, 26.3 (C-3′, C-4′), 17.2 (C-5′).

A solution of crude nitrile 25 in methanol (2 mL) was stirred in the presence of RANEY®-Ni under an atmosphere of hydrogen gas at ambient pressure. After completed reduction (30 min, CHCl_3_–MeOH 3 : 1 + 1 vol% NH_4_OH (25%)), the catalyst was filtered off and the filtrate was concentrated under reduced pressure. The residual oil was purified on silica gel (CHCl_3_–MeOH–NH_4_OH (25%) 8 : 4 : 1) to yield amine 26 (40.5 mg, 0.154 mmol, 49%, 2 steps) as a colourless oil. [*α*]^20^_D_: −30.2 (*c* = 1.2, MeOH); ^1^H-NMR (300 MHz, D_2_O) *δ* = 4.19 (dd, 1H, *J*_3,4_ 4.8 Hz, *J*_4,5_ 5.4 Hz, H-4), 4.09 (dd, 1H, *J*_1,2_ = *J*_2,3_ 7.0 Hz, H-2), 3.92 (dd, 1H, H-3), 3.82–3.68 (m, 2H, H-6), 2.89 (dd, 1H, *J*_1,5_ 7.8 Hz, H-1), 2.85 (t, 2H, H-6′), 2.58 (t, 2H, H-1′), 2.07–1.95 (m, 1H, H-5), 1.65–1.26 (m, 8H, H-2′, H-3′, H-4′, H-5′). ^13^C NMR (75.5 MHz, D_2_O) *δ* = 78.0 (C-3), 73.6 (C-2), 70.1 (C-4), 60.1 (C-6), 58.0 (C-1), 47.5, 47.5 (C-5, C-1′), 39.8 (C-6′), 28.3, 28.2, 26.0, 25.5 (C-2′, C-3′, C-4′, C-5′). MS (MALDI): calculated for [C_12_H_26_N_2_O_4_H]: *m*/*z* 263.1971 [M + H]^+^; found [M + H]^+^ 263.1971.

#### 5-(Dimethylamino)-*N*-(6-(((1*S*,2*S*,3*S*,4*S*,5*R*)-2,3,4-trihydroxy-5-(hydroxymethyl)cyclopentyl)amino)hexyl)naphthalene-1-sulfonamide or 1-(6-dansylaminohexyl)amino-α-d-*galacto*-cyclopentane (27)

Amine 26 (38.7 mg, 0.115 mmol) was dissolved in methanol and Na_2_CO_3_ (24.5 mg, 0.231 mmol) and dansyl chloride (34.2 mg, 127 mmol) were added. After completed reaction (15 min, CHCl_3_–MeOH 3 : 1 + 1 vol% NH_4_OH (25%)) the suspension was concentrated under reduced pressure. The residue was purified on silica gel (CHCl_3_–MeOH 8 : 1 + 1 vol% NH_4_OH (25%)) to provide dansyl amide 27 (31.2 mg, 62.9 μmol, 55%) as a yellow syrup. [*α*]^20^_D_: −6.7 (*c* = 0.92, MeOH); ^1^H-NMR (300 MHz, CD_3_OD) *δ* = 8.56 (d, 1H, dansyl), 8.36 (d, 1H, dansyl), 8.19 (d, 1H, dansyl), 7.63–7.54 (m, 2H, dansyl), 7.28 (d, 1H, dansyl), 4.32 (dd, 1H, *J*_3,4_ 4.3 Hz, *J*_4,5_ 7.1 Hz, H-4), 4.16 (dd, 1H, *J*_1,2_ = 6.5 Hz, *J*_2,3_ 3.8 Hz, H-2), 3.94 (dd, 1H, H-3), 3.86 (dd, 1H, *J*_5,6a_ 5.6 Hz, *J*_6a,6b_ 10.8 Hz, H-6a), 3.78 (dd, 1H, *J*_5,6a_ 7.0 Hz, H-6b), 3.57 (dd, 1H, *J*_1,5_ 7.7 Hz, H-1), 2.98 (t, 2H, H-1′), 2.89 (s, 6H, dansyl), 2.84 (t, 2H, H-6′), 2.32 (dddd, 1H, H-5), 1.65–1.14 (m, 8H, H-2′, H-3′, H-4′, H-5′). ^13^C NMR (75.5 MHz, CD_3_OD) *δ* = 153.2, 137.2 (ipso), 131.3, 131.1, 131.0, 130.1, 129.1, 124.3, 120.6, 119.5 (dansyl), 79.3 (C-3), 73.3 (C-2), 72.1 (C-4), 61.9 (C-1), 61.4 (C-6), 47.8 (C-1′), 46.4 (C-5), 45.8 (dansyl), 43.6 (C-6′), 30.4, 27.0, 26.9, 26.9 (C-2′, C-3′, C-4′, C-5′). MS (MALDI): calculated for [C_24_H_37_N_3_O_6_SH]: *m*/*z* 496.2481 [M + H]^+^; found [M + H]^+^ 496.2481.

## Author contributions

P. W. performed the synthesis, M. T. and A. W. assisted with the development and the syntheses. R. F. provided crystal structure for compound 21. S. A. N. performed biochemical experiments and S. G. W. supervised and evaluated biochemical studies. P. W. and A. E. S. conceived and designed the synthetic experiments. P. W., S. A. N., A. E. S., S. G. W. and T. M. W. prepared and reviewed the manuscript. All authors have read and agreed to the published version of the manuscript.

## Conflicts of interest

There are no conflicts to declare.

## Supplementary Material

RA-011-D1RA02507D-s001

RA-011-D1RA02507D-s002
