# Supplementary material for: New α-galactosidase-inhibiting aminohydroxycyclopentanes
Source: RSC Adv. 2021 Apr 29;11(26):15943–51. doi: 10.1039/d1ra02507d (PMC9029992; doi:10.1039/d1ra02507d)

Electronic Supplementary Material (ESI) for RSC Advances.
This journal is © The Royal Society of Chemistry 2021

Supplementary Materials:

New α-galactosidase-inhibiting aminohydroxycyclopentanes

Patrick Weber,*[a] Roland Fischer,[b] Seyed A. Nasseri,[c] Arnold E. Stütz,[a] Martin Thonhofer,[a] Stephen G. Withers,[c] Andreas Wolfsgruber,[a] and Tanja M. Wrodnigg[a]

[a]*Glycogroup, Institute of Chemistry and Technology of Biobased Systems, Graz University of Technology, Stremayrgasse 9, A-8010 Graz, Austria*

[b]*Institute of Inorganic Chemistry, Graz University of Technology, Stremayrgasse 9, A-8010 Graz, Austria*

[c]*Chemistry Department, University of British Columbia, 2036 Main Mall, Vancouver, BC, Canada V6T 1Z1*

Supplementary data: ^{1}H NMR, ^{13}C NMR, COSY, and HSQC spectra

(3a*S*,3b*S*,6a*S*,7*S*,7a*S*)-1-Benzyl-5,5-dimethylhexahydro-1H-[1,3]dioxolo [4',5':3,4]cyclopenta[1,2-*c*]isoxazol-7-ol (16)

^{1}H NMR (300 MHz, $CDCl_3$): Compound **16**

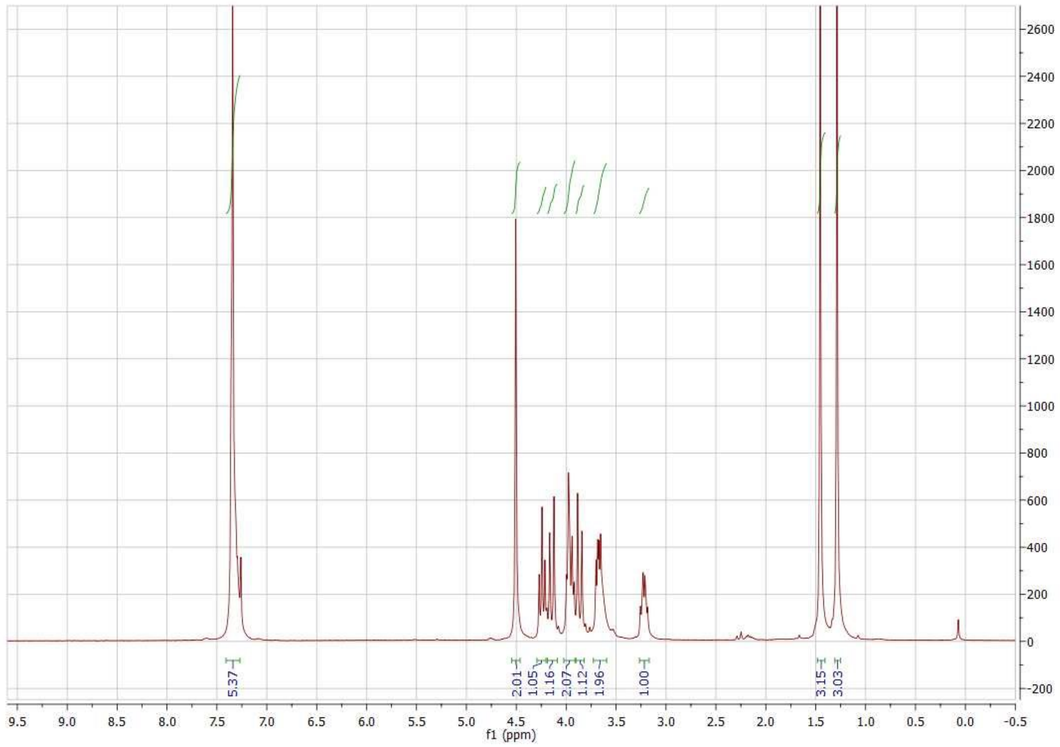

^{13}C NMR (75.5 MHz, $CDCl_3$): Compound **16**

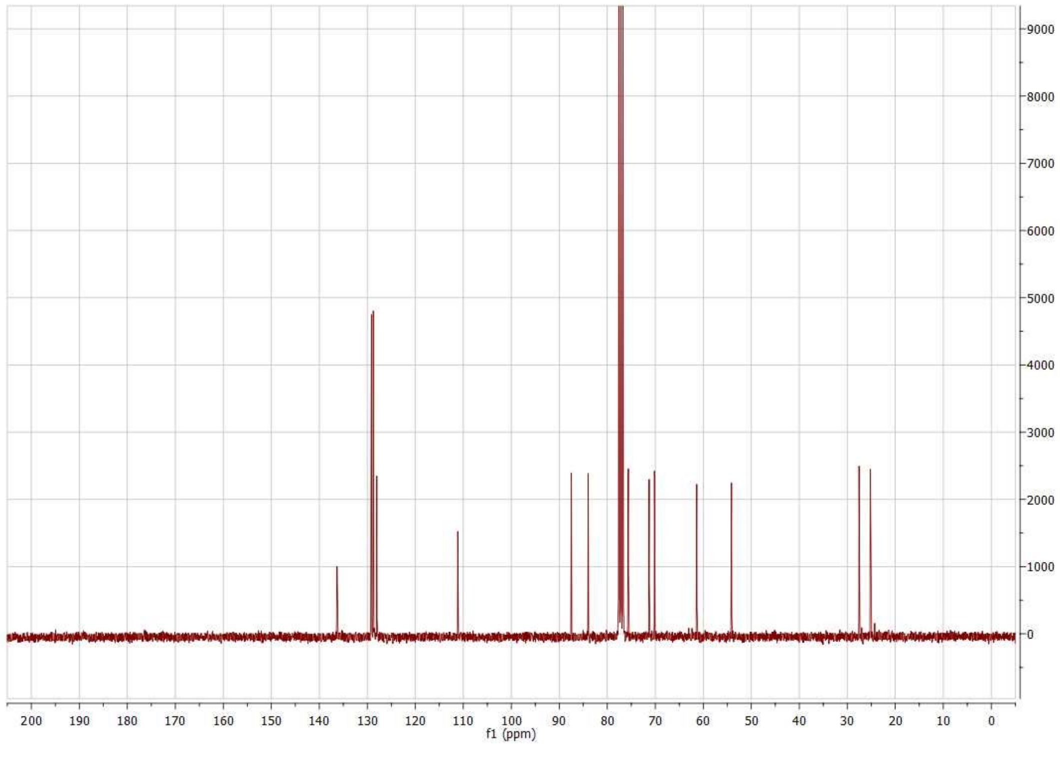

COSY ($CDCl_3$): Compound **16**

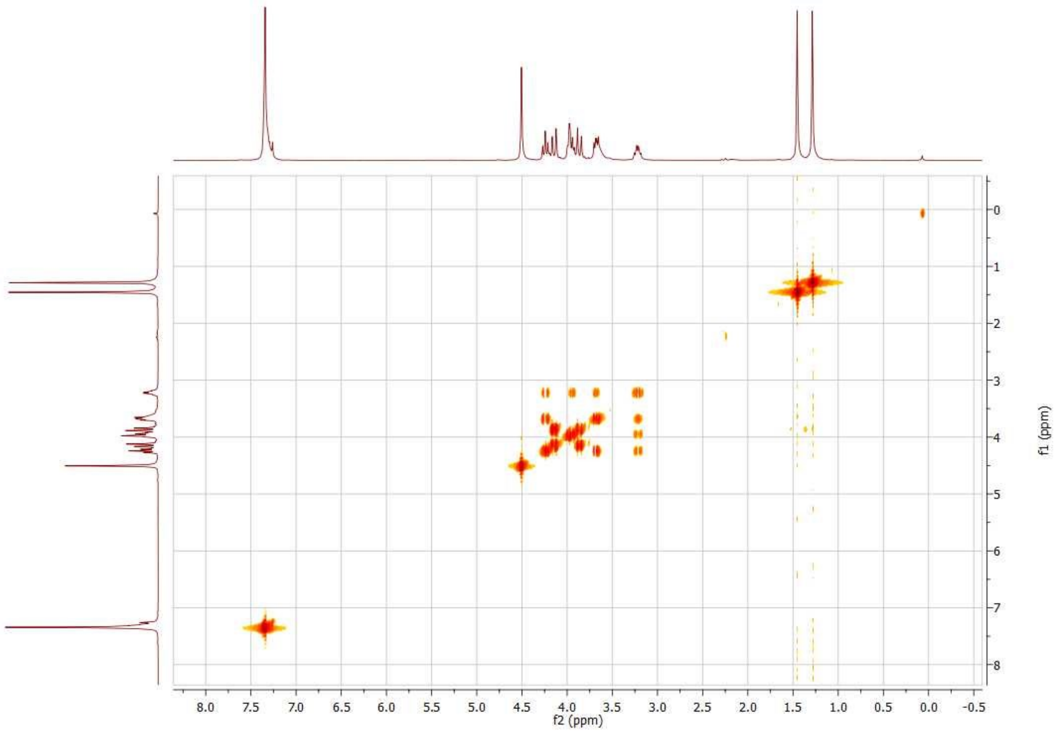

HSQC ($CDCl_3$): Compound **16**

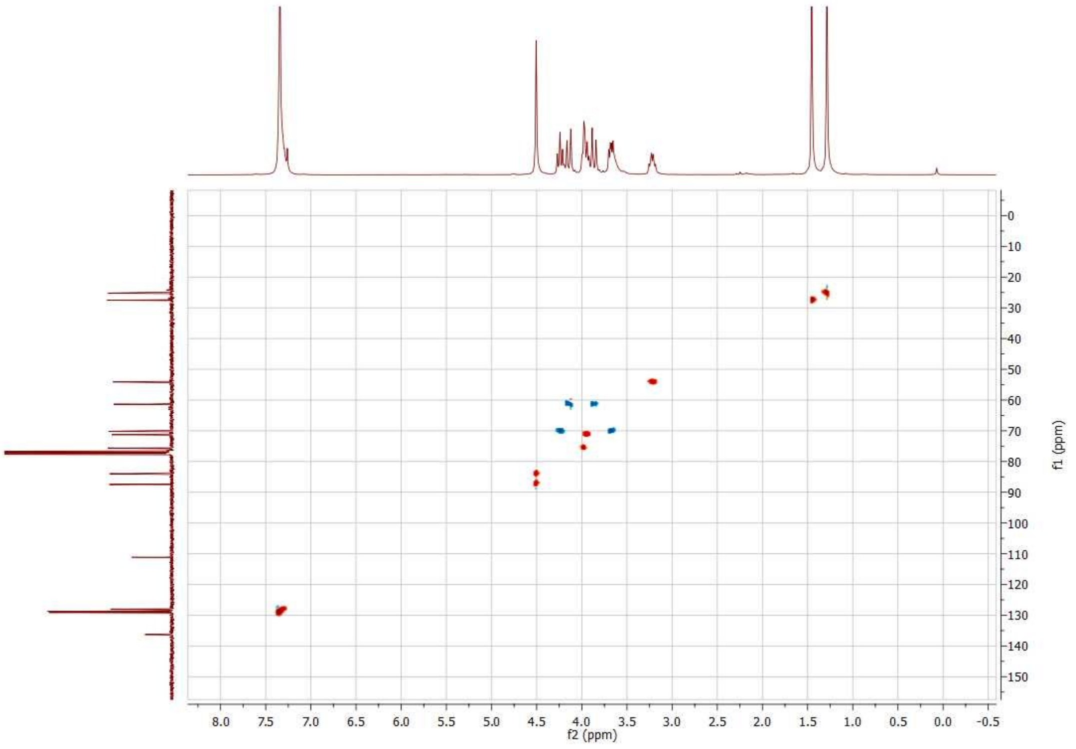

(3a*S*,3b*S*,6a*S*,7*S*,7a*S*)-1-Benzyl-7-(methoxymethoxy)-5,5-dimethylhexahydro-1H-[*1,3*]dioxolo[*4',5':3,4*]cyclopenta[*1,2-c*]isoxazole (17)

^{1}H NMR (300 MHz, $CDCl_3$): Compound **17**

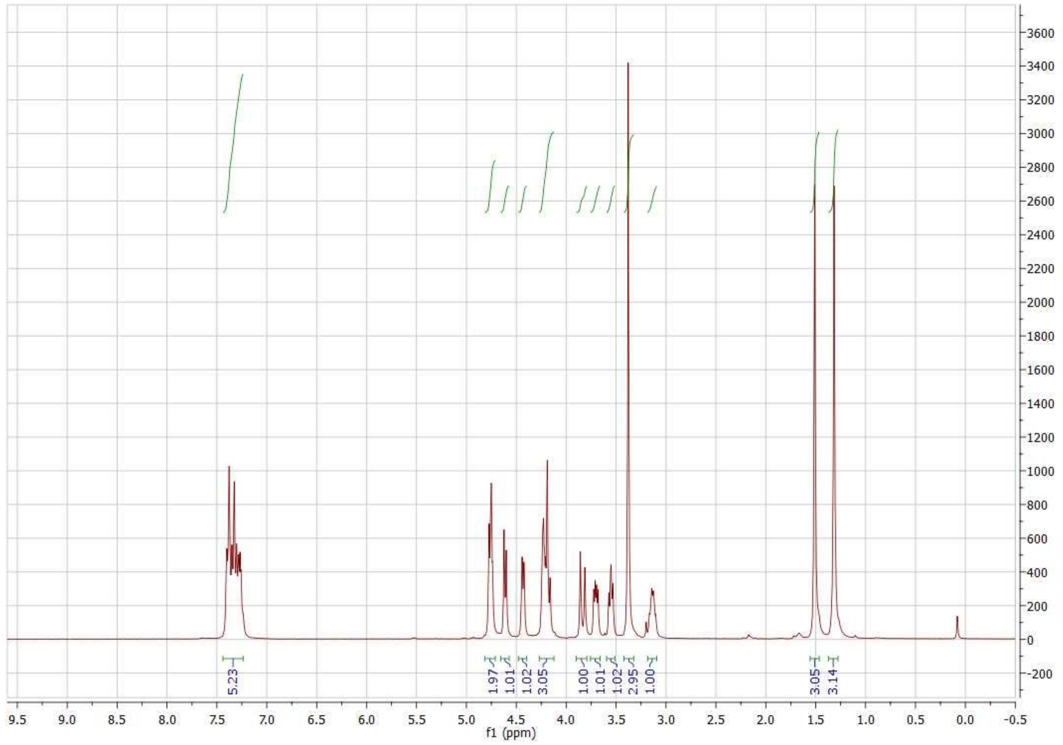

^{13}C NMR (75.5 MHz, $CDCl_3$): Compound **17**

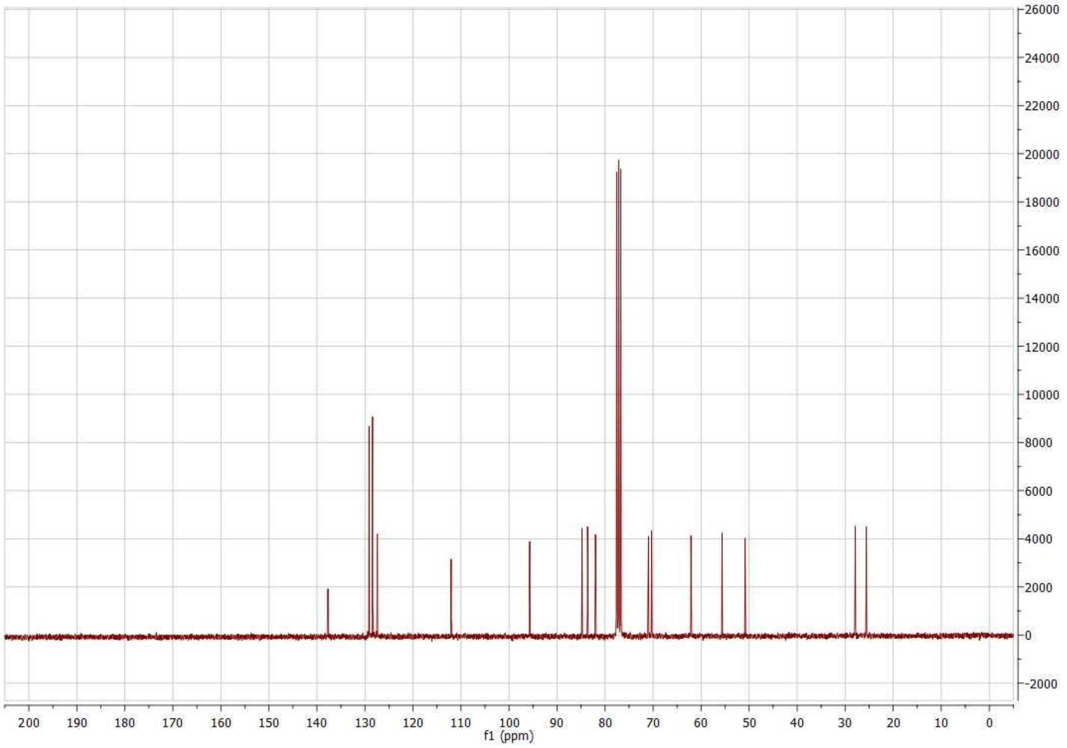

COSY ($CDCl_3$): Compound **17**

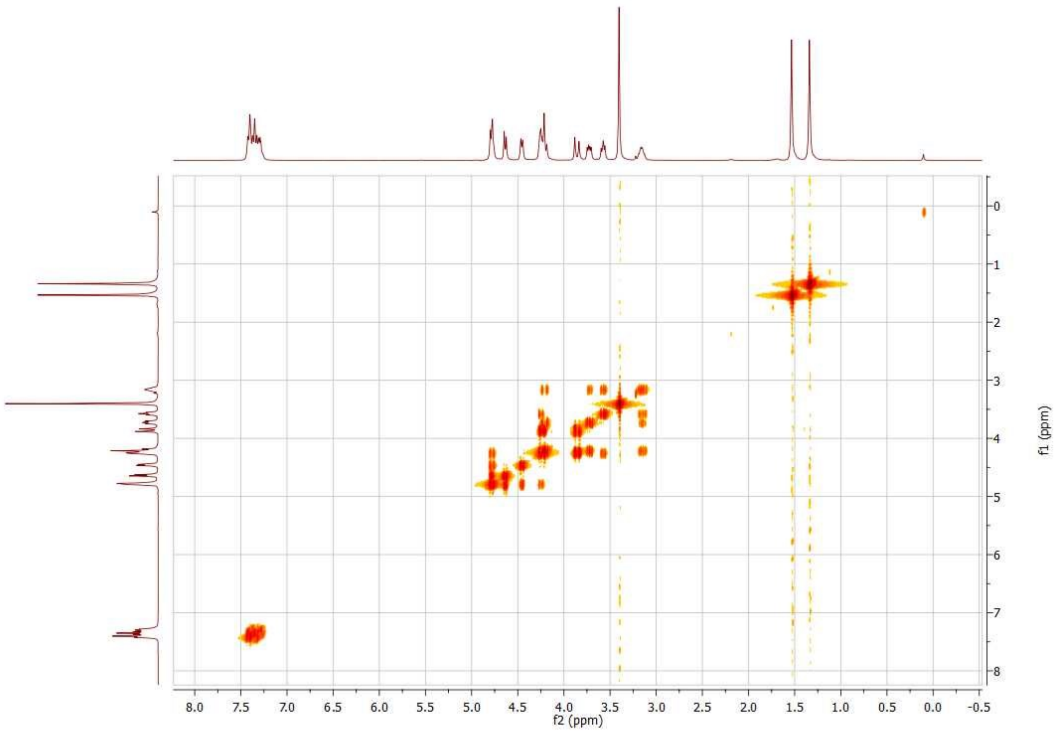

HSQC ($CDCl_3$): Compound **17**

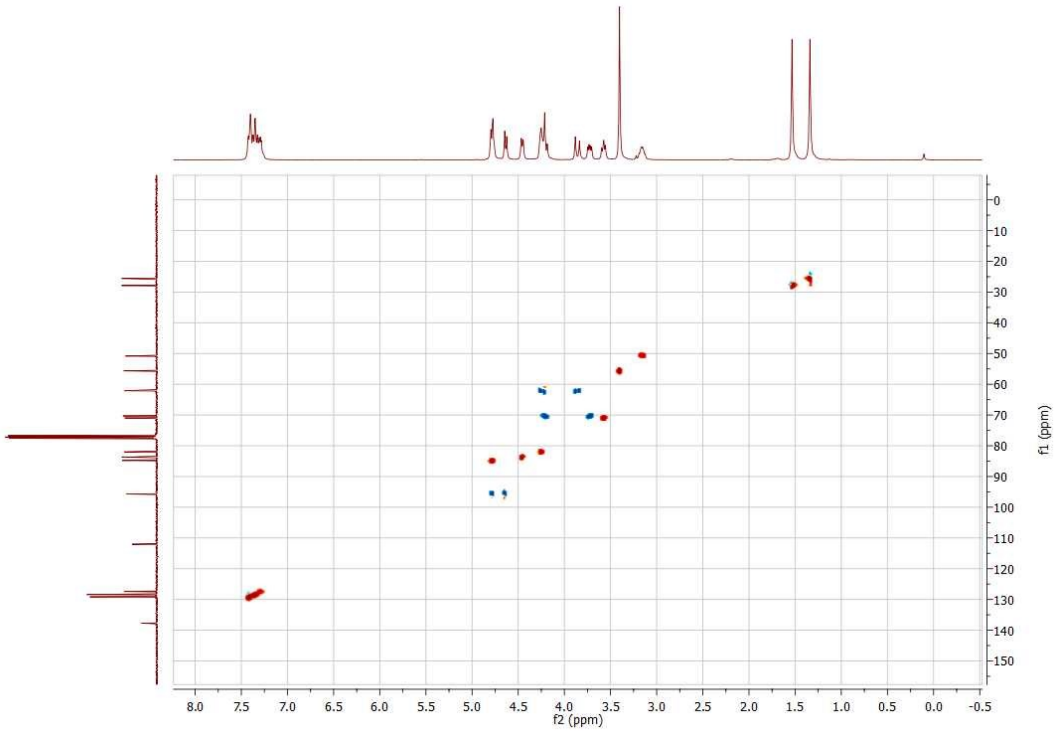

tert-Butyl [(3a_S_,4_S_,5_S_,6_S_,6a_S_)-4-(hydroxymethyl)-6-(methoxymethoxy)-2,2-dimethyl tetrahydro-4H-cyclopenta[_d_][_1,3_]dioxol-5-yl]carbamate or **1-(_tert_-Butyloxycarbonyl) amino-3,4-_O_-isopropylidene-2-_O_-methoxymethyl-β-L-_altro_-cyclopentane (18)**

^{1}H NMR (300 MHz, $CDCl_3$): Compound **18**

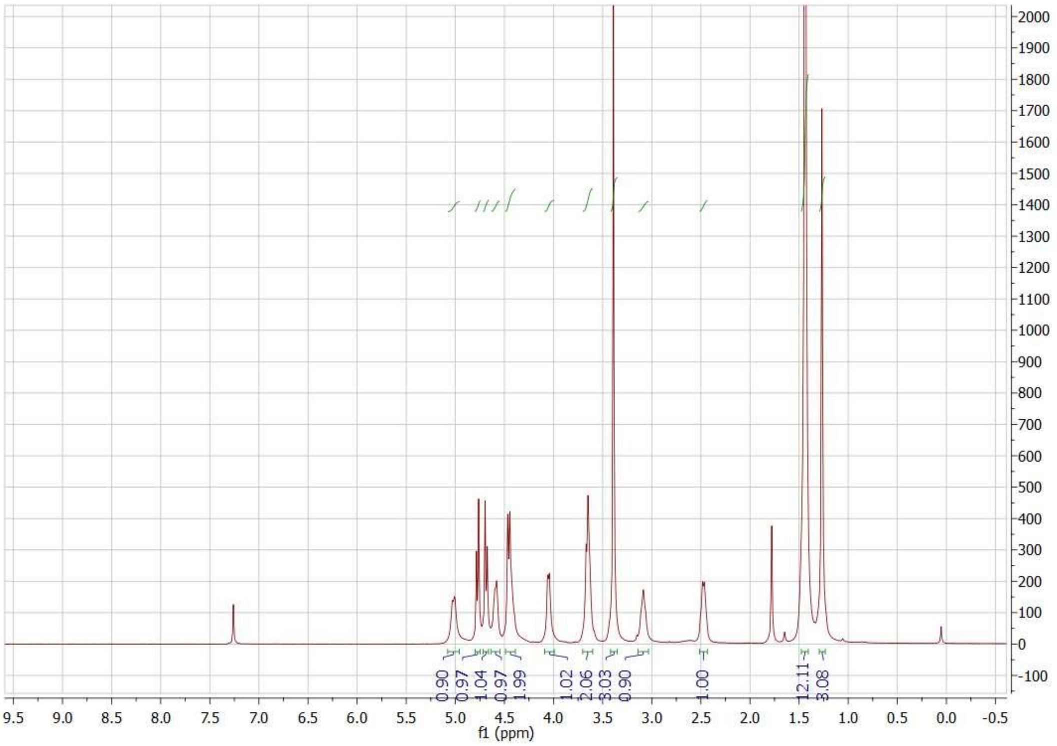

^{13}C NMR (75.5 MHz, $CDCl_3$): Compound **18**

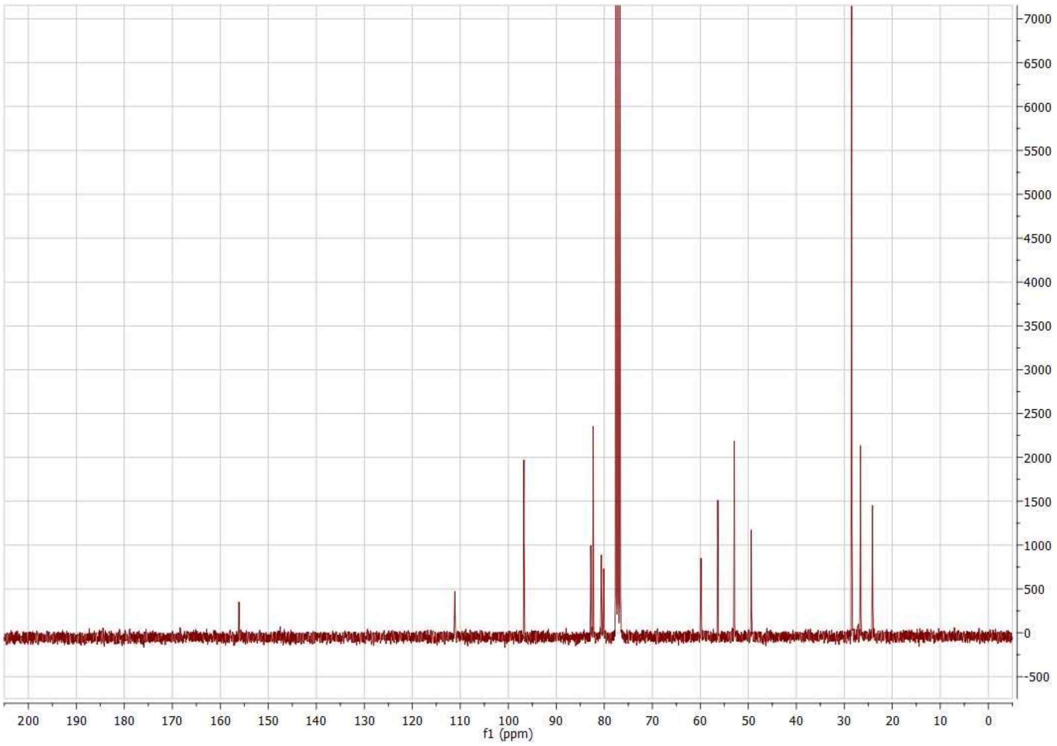

COSY ($CDCl_3$): Compound **18**

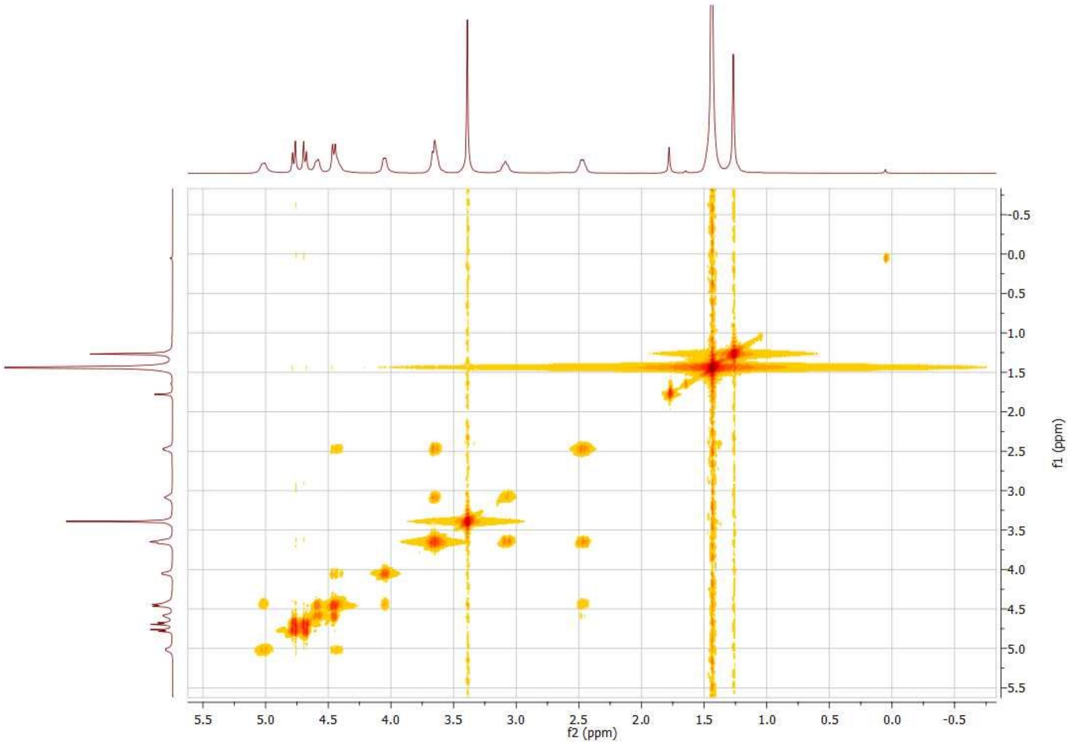

HSQC ($CDCl_3$): Compound **18**

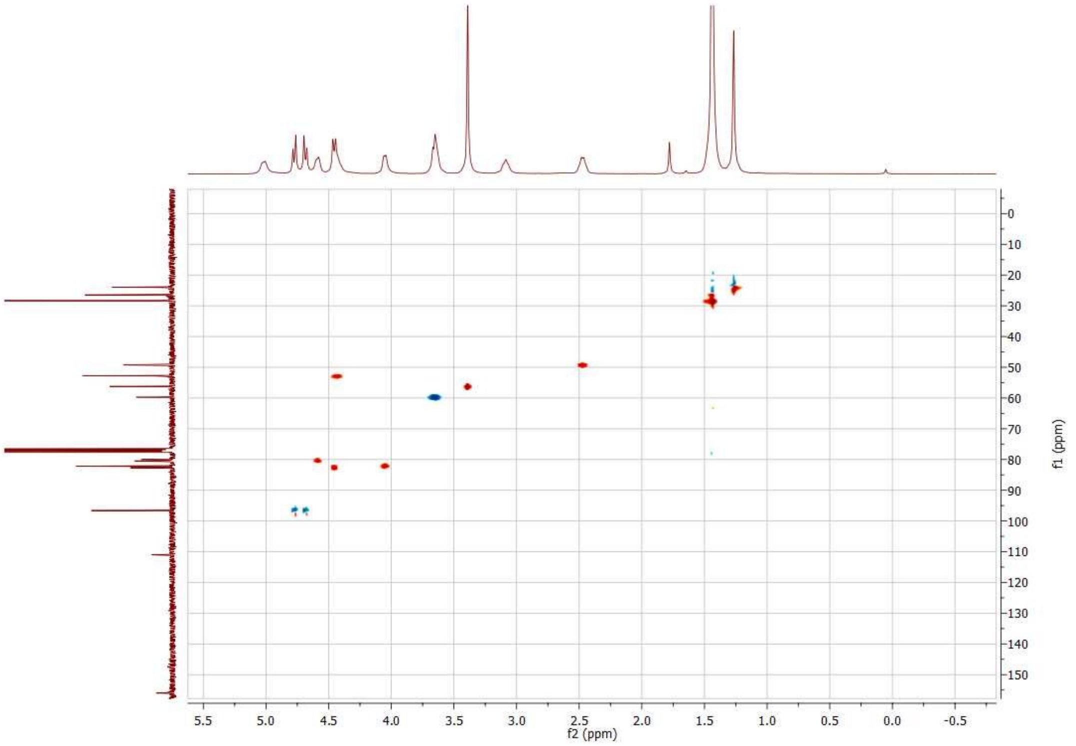

tert-Butyl [(3a*S*,4*R*,5*S*,6*S*,6a*S*)-4-(hydroxymethyl)-6-(methoxymethoxy)-2,2-dimethyl tetrahydro-4H-cyclopenta[*d*][*1,3*]dioxol-5-yl]carbamate or **1-(*tert*-Butyloxycarbonyl) amino-3,4-*O*-isopropylidene-2-*O*-methoxymethyl-α-D-*galacto*-cyclopentane (21)**

^{1}H NMR (300 MHz, $CDCl_3$): Compound **21**

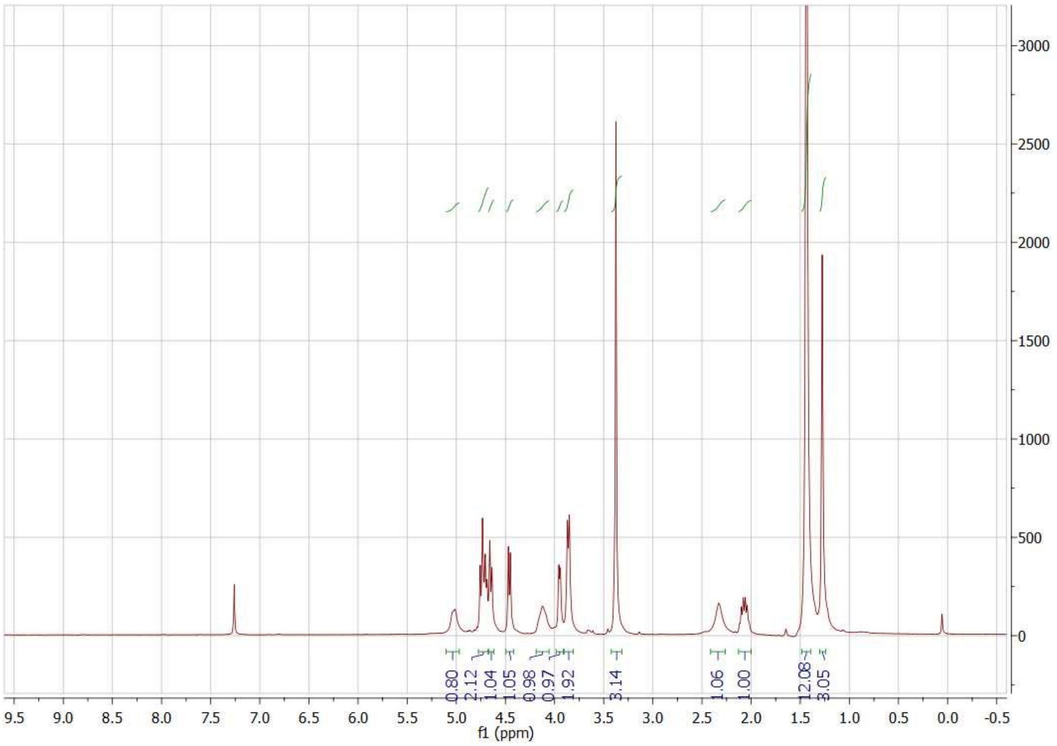

^{13}C NMR (75.5 MHz, $CDCl_3$): Compound **21**

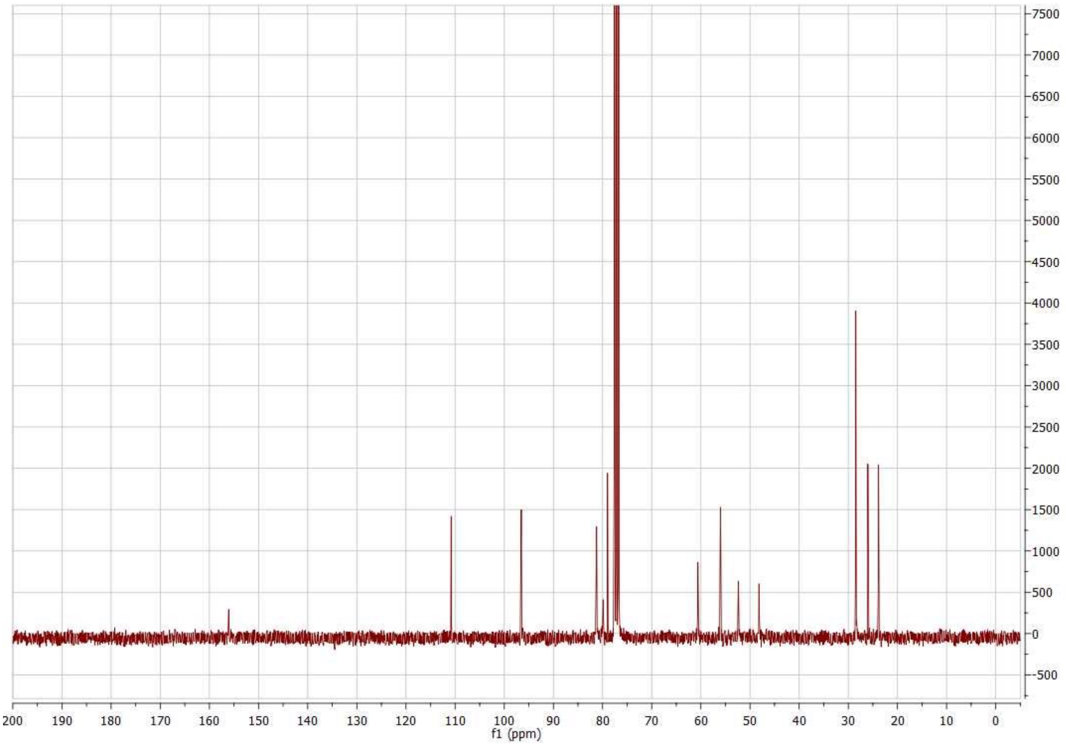

COSY ($CDCl_3$): Compound **21**

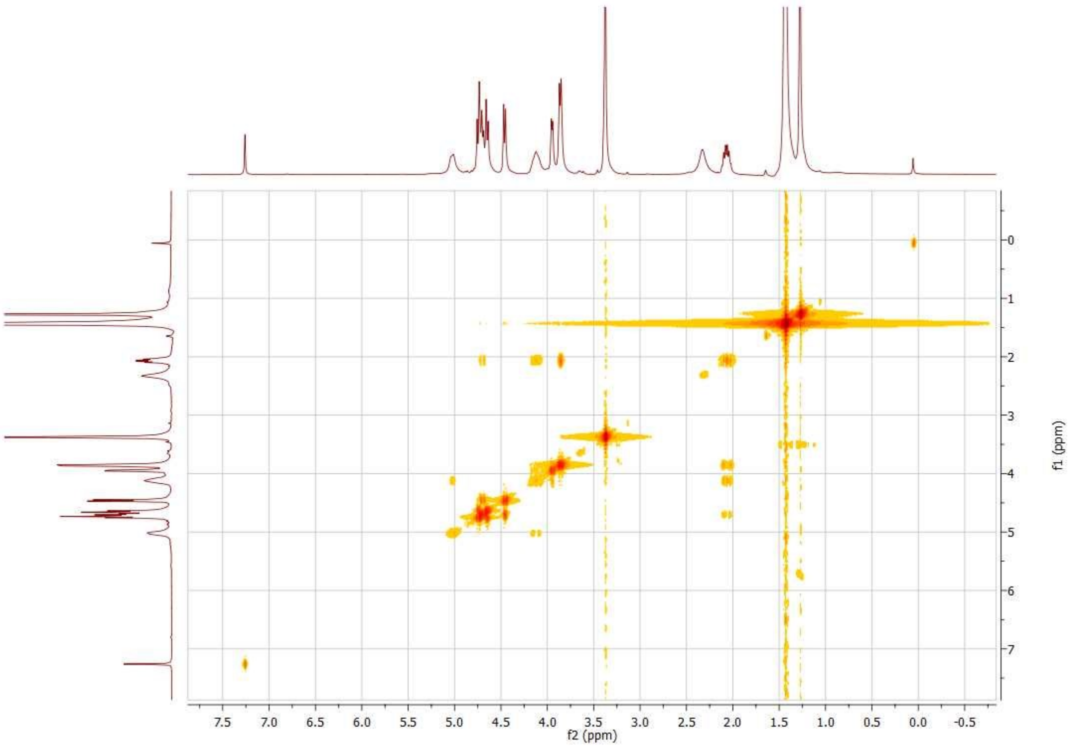

HSQC ($CDCl_3$): Compound **21**

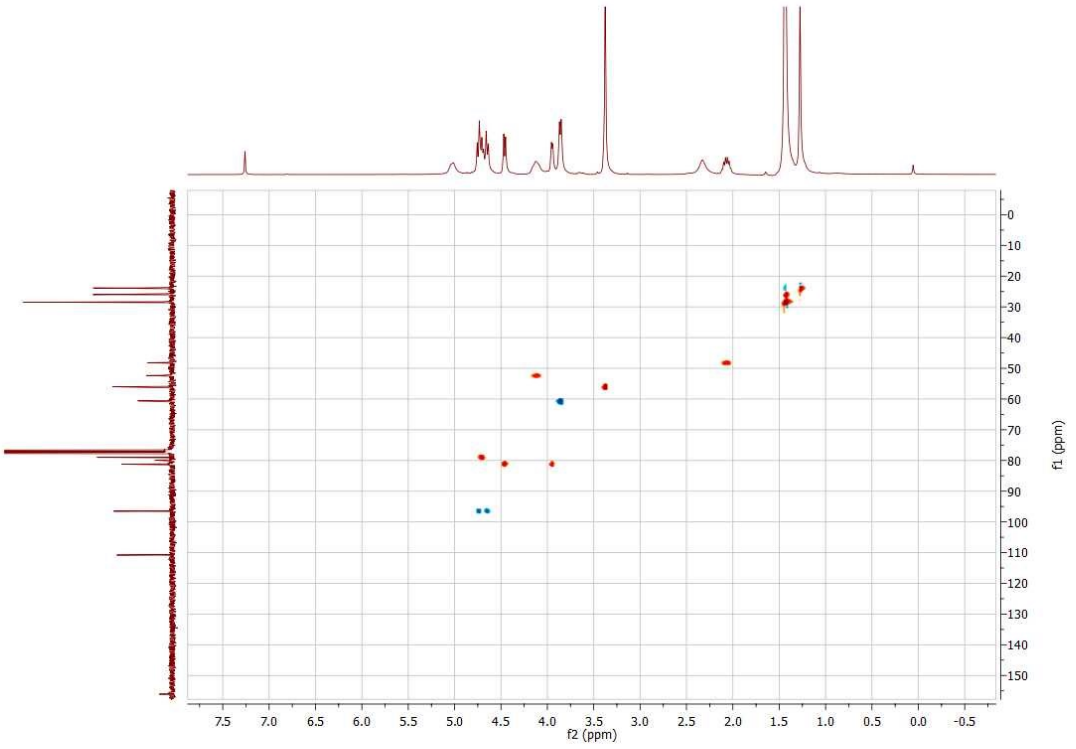

(1*S*,2*S*,3*S*,4*S*,5*R*)-4-Amino-5-(hydroxymethyl)cyclopentane-1,2,3-triol or 1-Amino-α-D-*galacto*-cyclopentane (22)

^{1}H NMR (300 MHz, D_2O, free base): Compound **22**

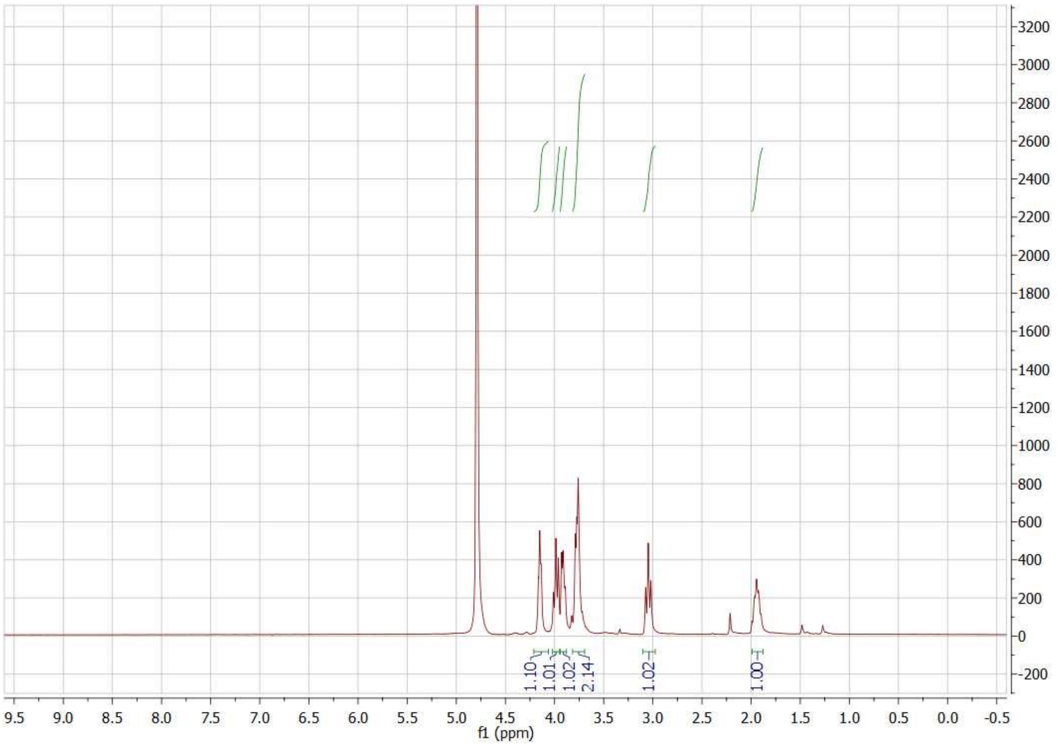

^{13}C NMR (75.5 MHz, D_2O, free base): Compound **22**

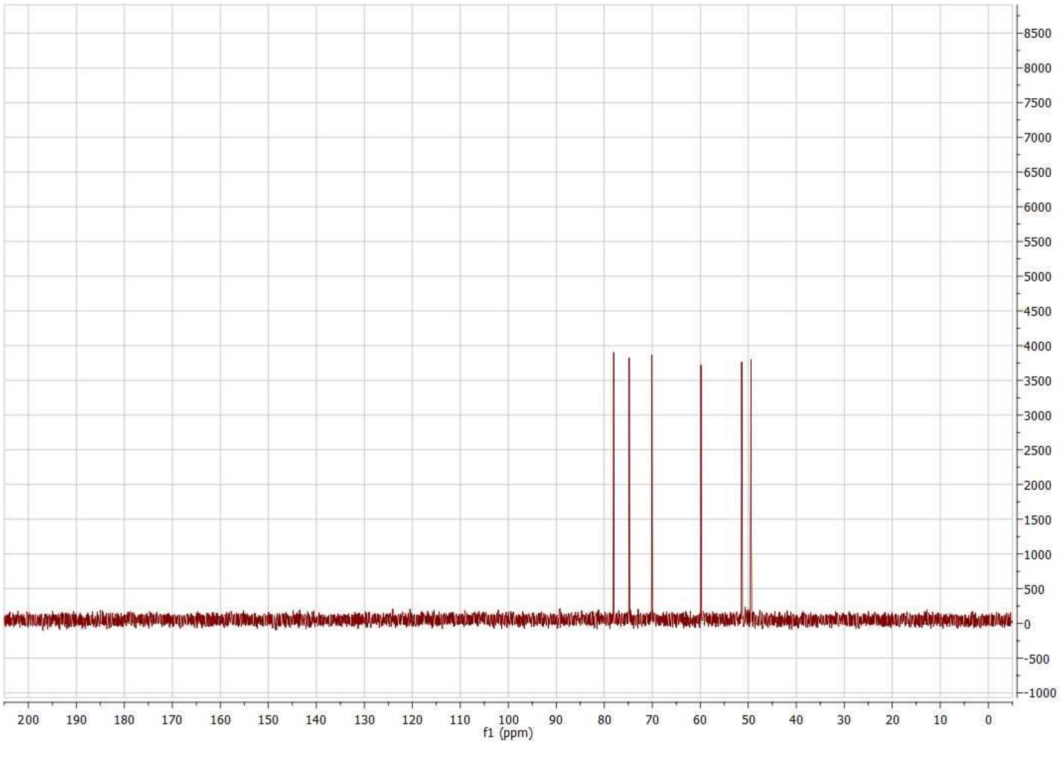

COSY (D_2O, free base): Compound **22**

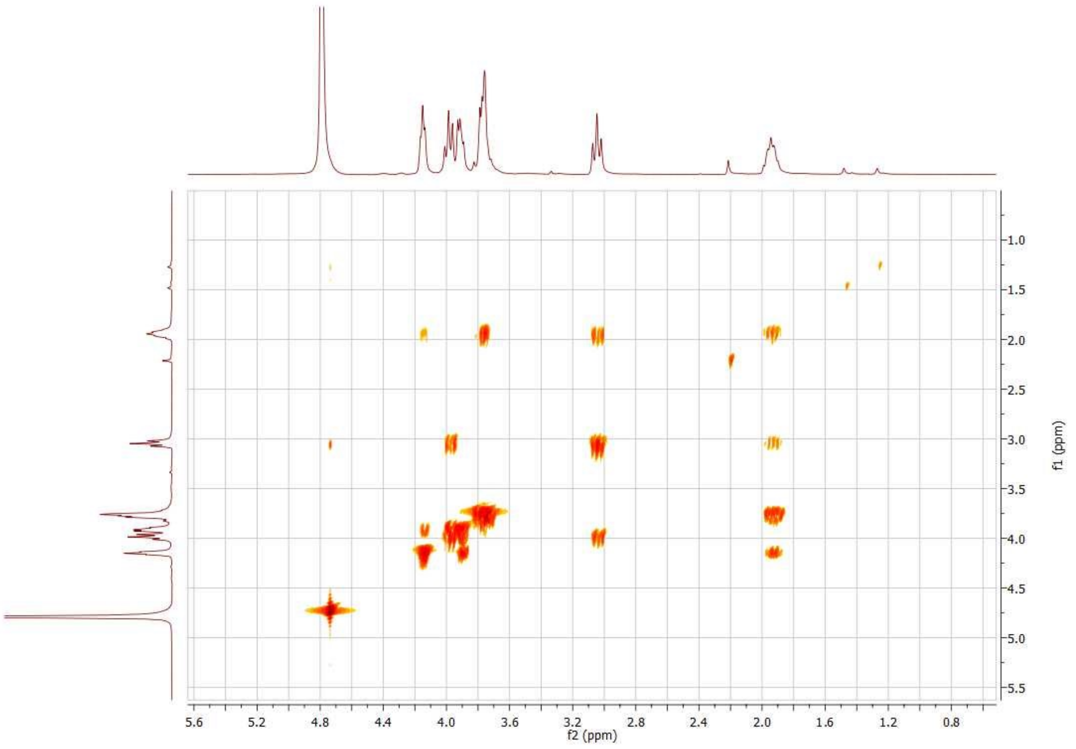

HSQC (D_2O, free base): Compound **22**

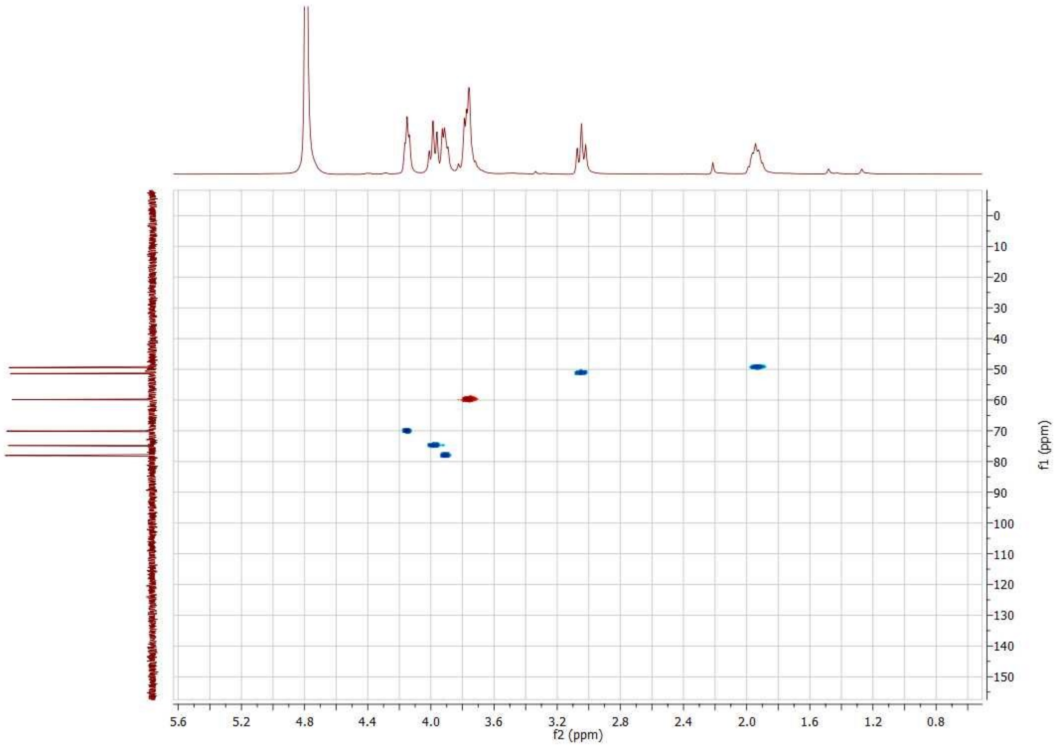

¹H NMR (300 MHz, D_2O, hydrochloride): Compound **22**·HCl

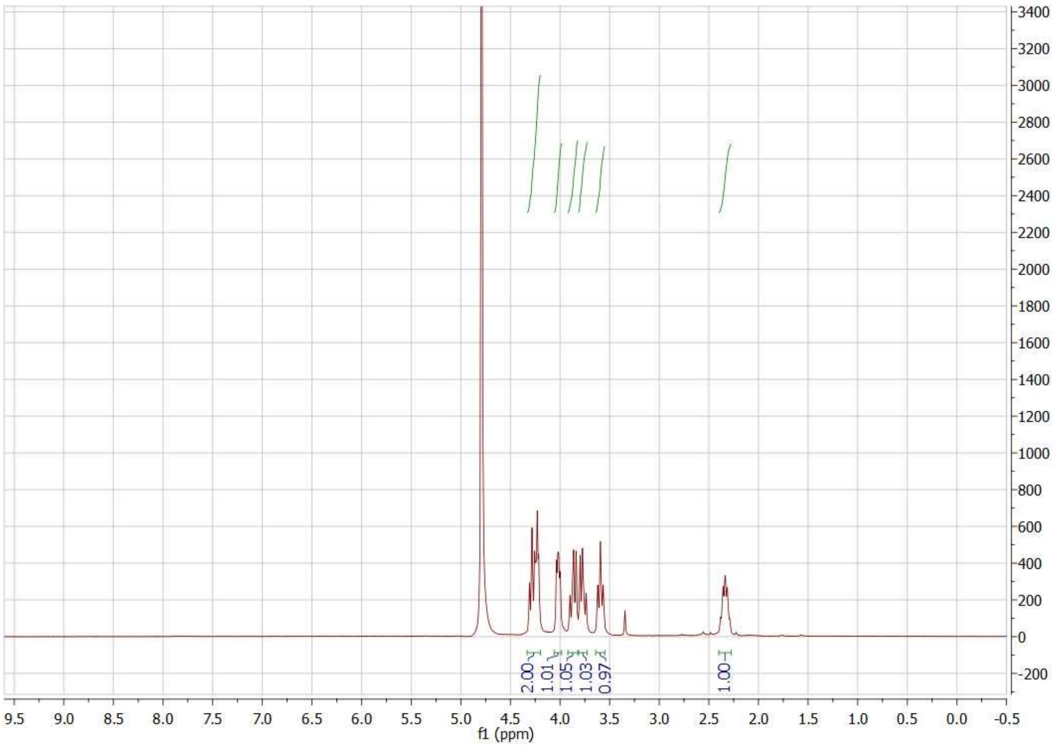

¹³C NMR (75.5 MHz, D_2O, hydrochloride): Compound **22**·HCl

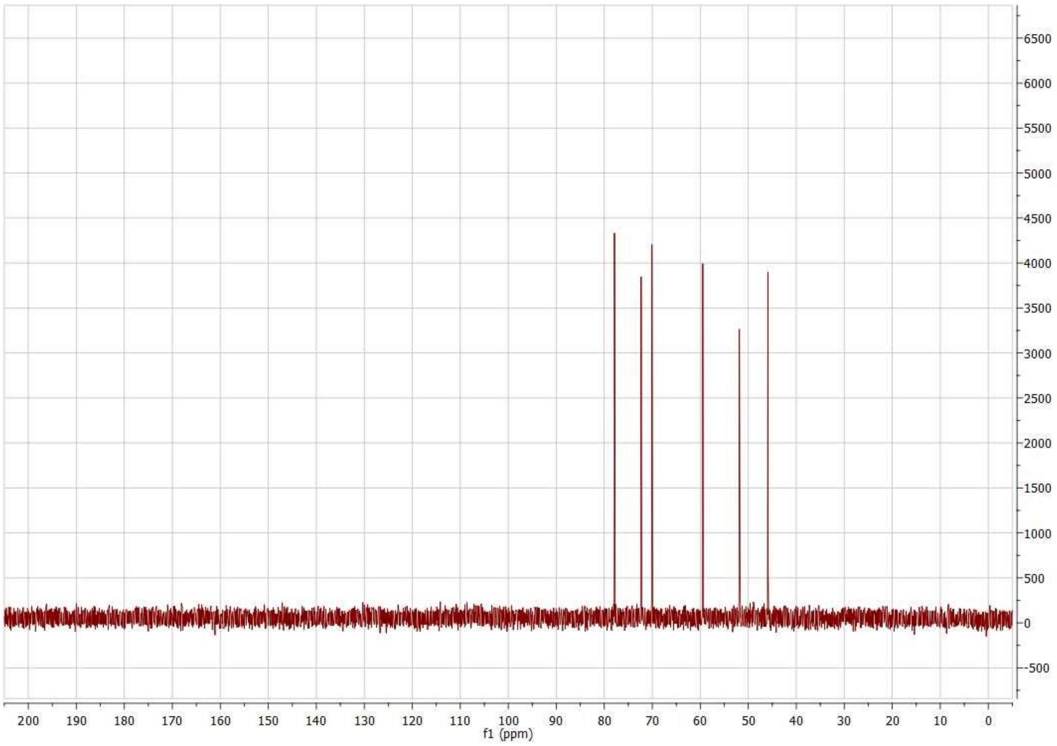

COSY (D_2O, hydrochloride): Compound **22**·HCl

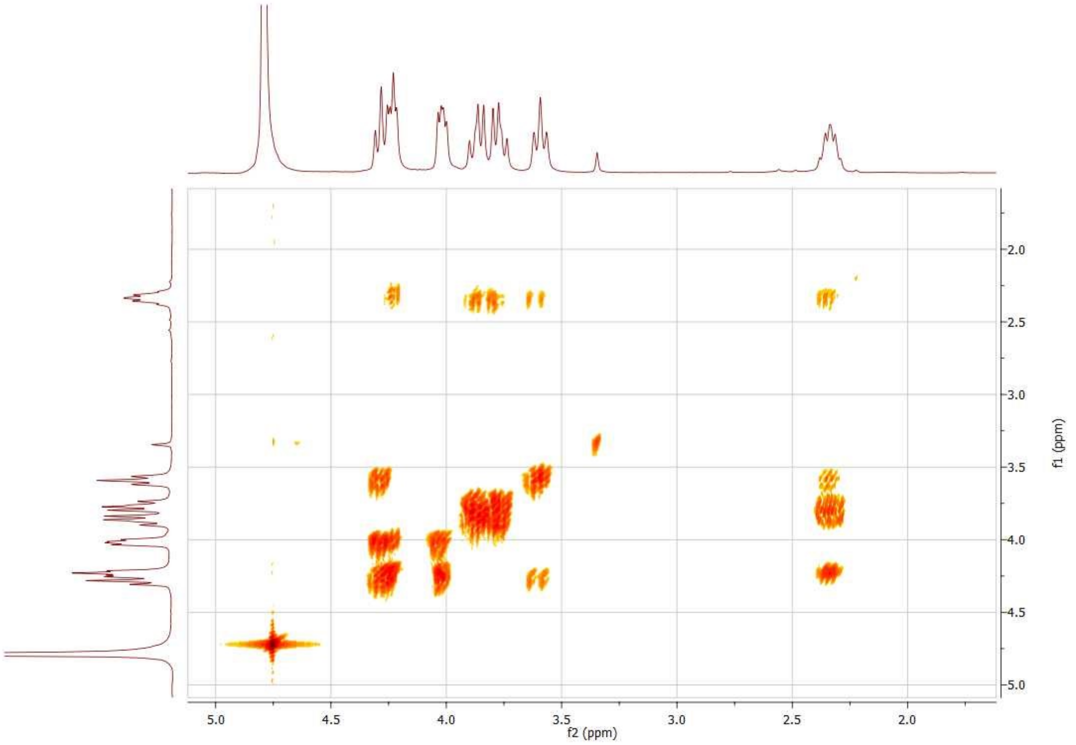

HSQC (D_2O, hydrochloride): Compound **22**·HCl

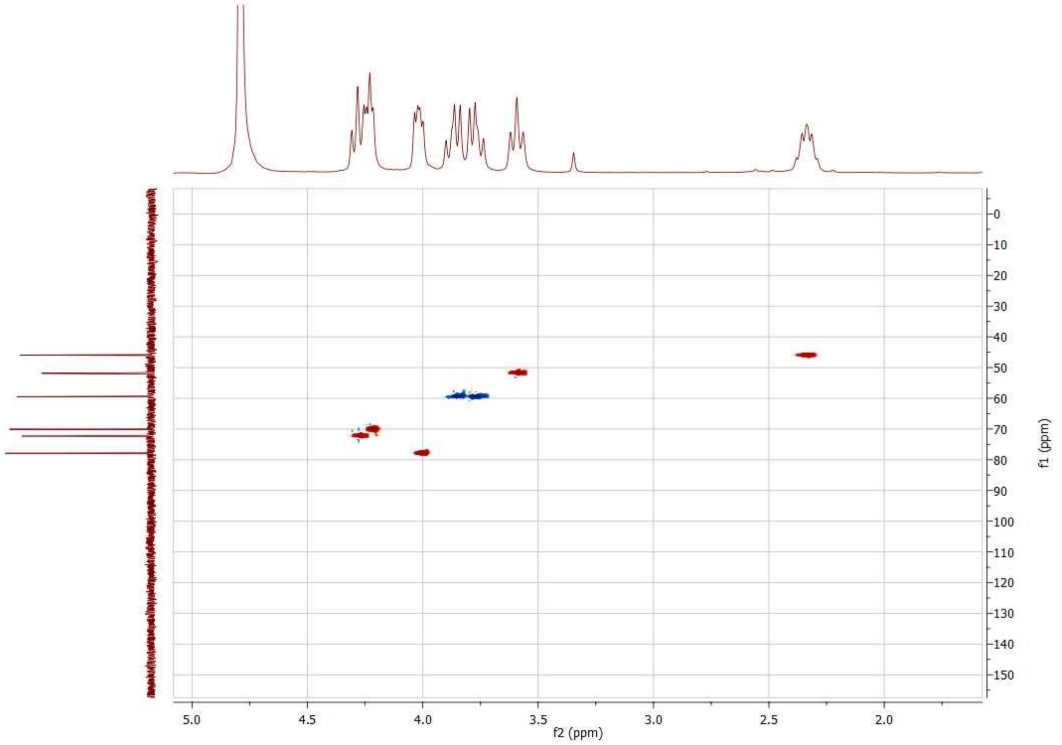

(1*S*,2*S*,3*S*,4*S*,5*R*)-4-(*n*-Hexylamino)-5-(hydroxymethyl)cyclopentane-1,2,3-triol or **1-(Hexyl)amino-α-D-*galacto*-cyclopentane (23)**

^{1}H NMR (300 MHz, CD_3OD): Compound **23**

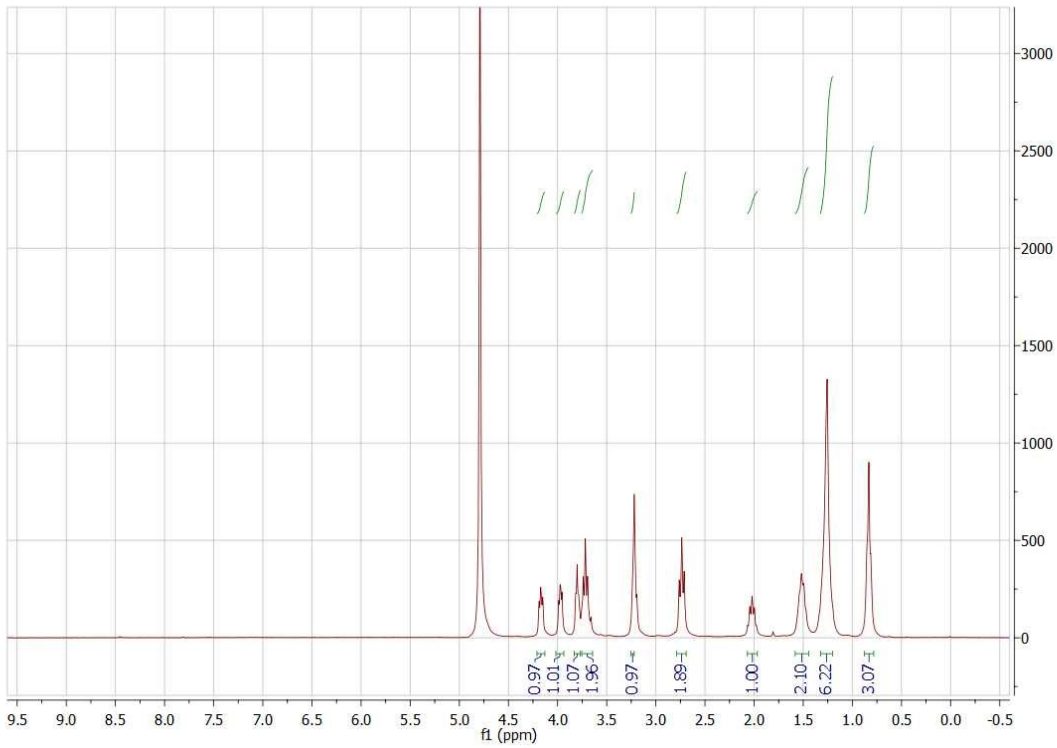

^{13}C NMR (75.5 MHz, CD_3OD): Compound **23**

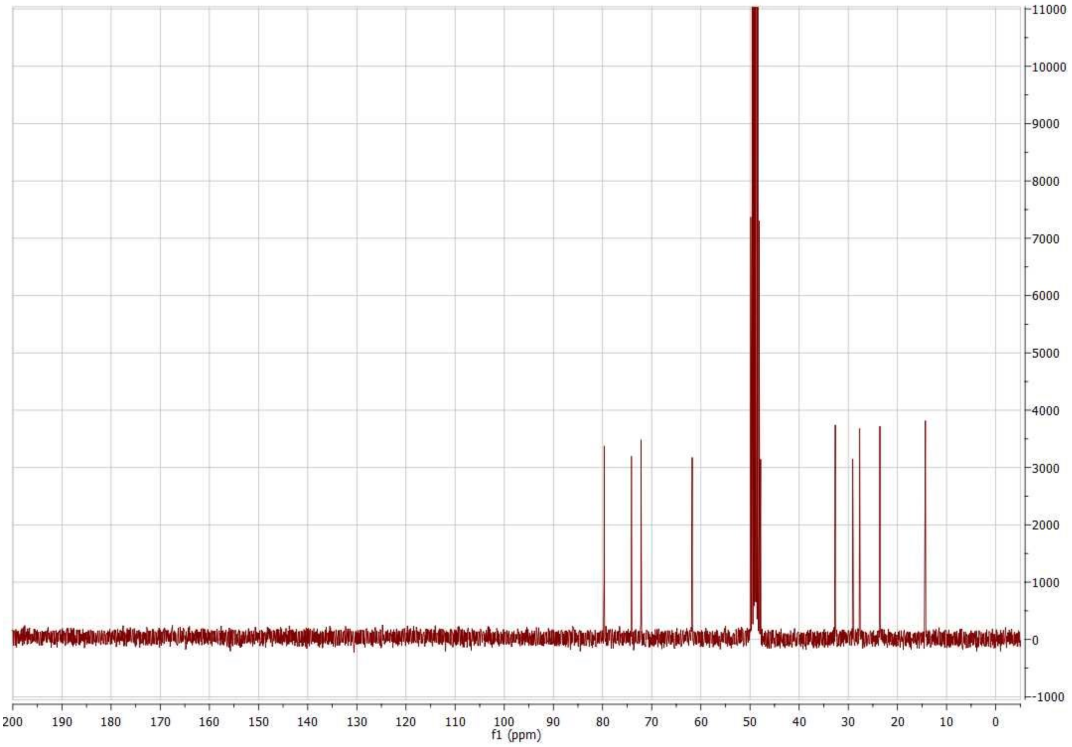

COSY (CD_3OD): Compound **23**

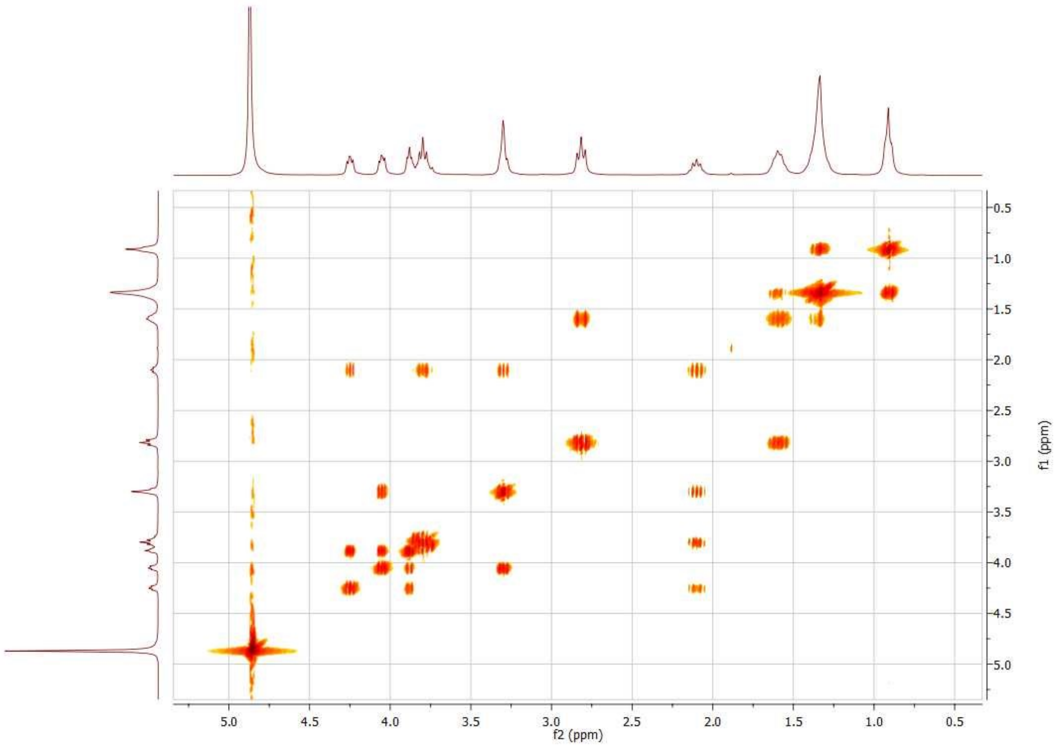

HSQC (CD_3OD): Compound **23**

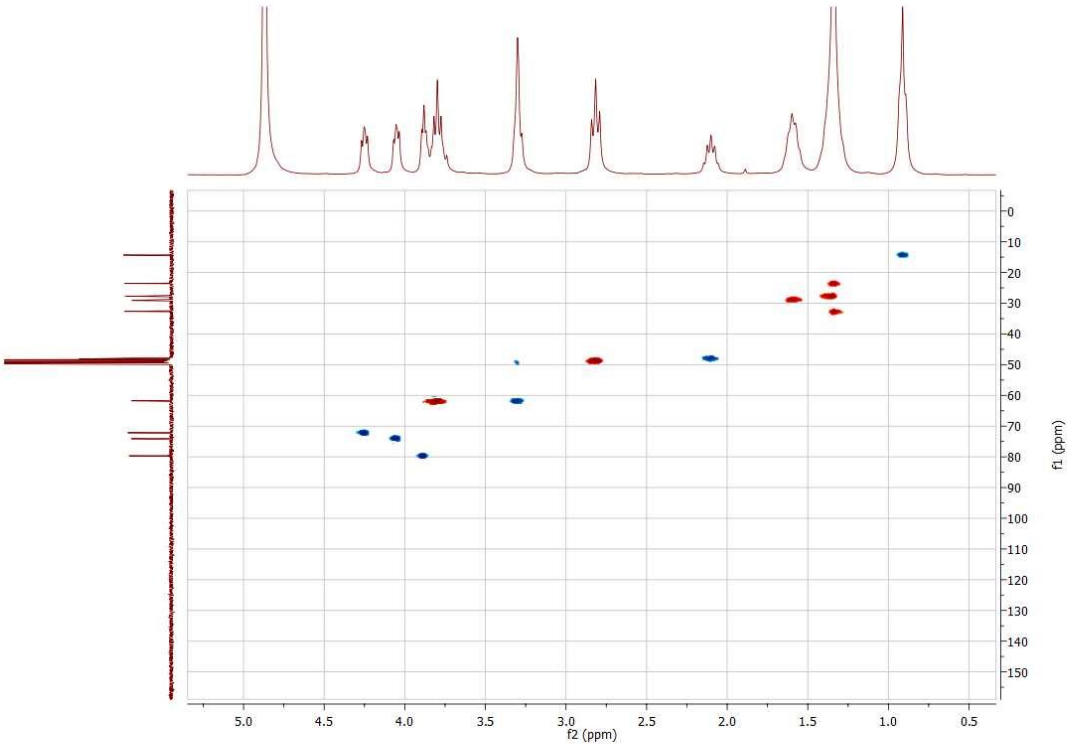

(1*S*,2*S*,3*S*,4*S*,5*R*)-4-(*n*-Nonylamino)-5-(hydroxymethyl)cyclopentane-1,2,3-triol or **1-(Nonyl)amino-α-D-*galacto*-cyclopentane (24)**

^{1}H NMR (300 MHz, CD_3OD): Compound **24**

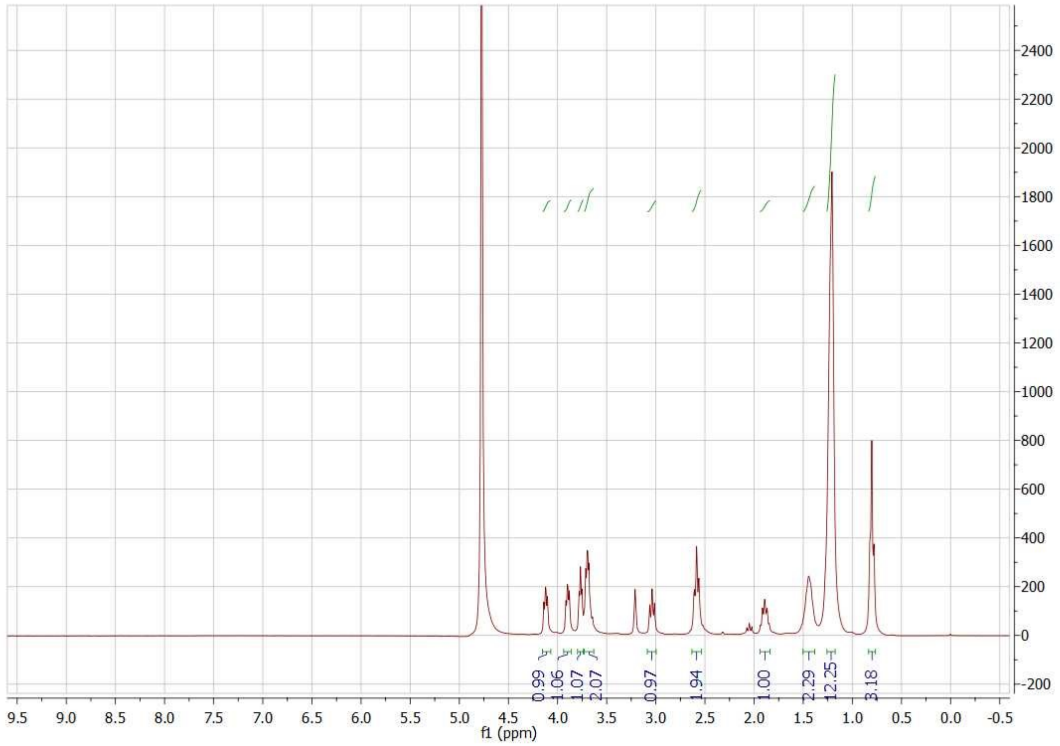

^{13}C NMR (75.5 MHz, CD_3OD): Compound **24**

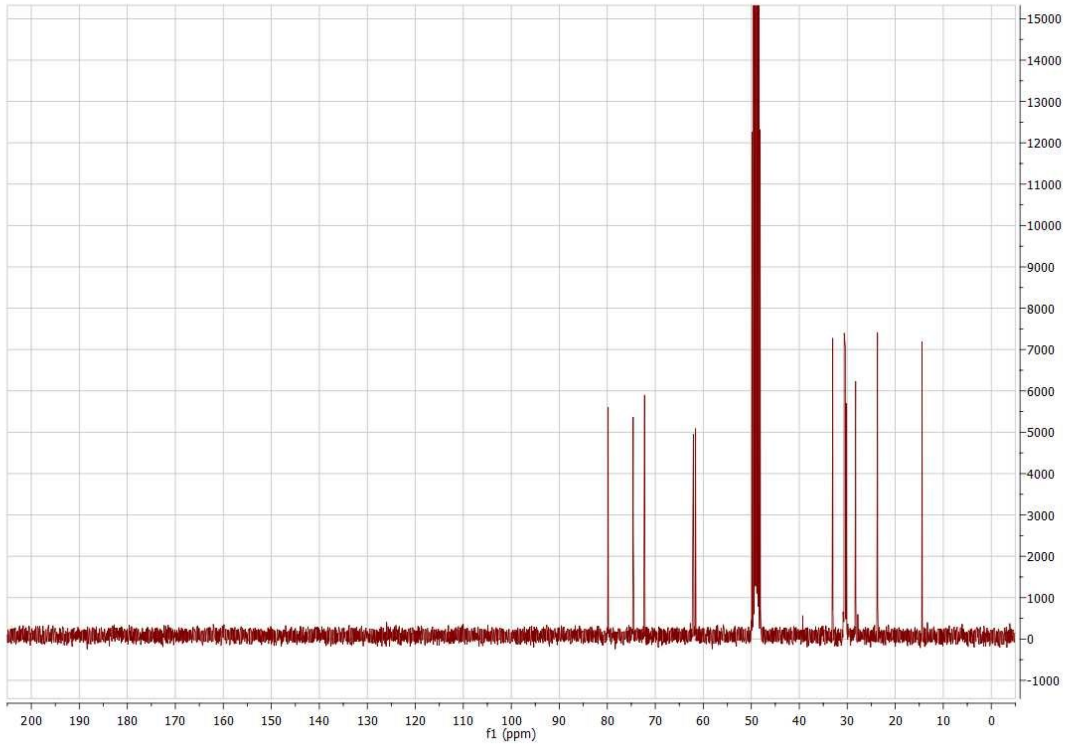

COSY (CD_3OD): Compound **24**

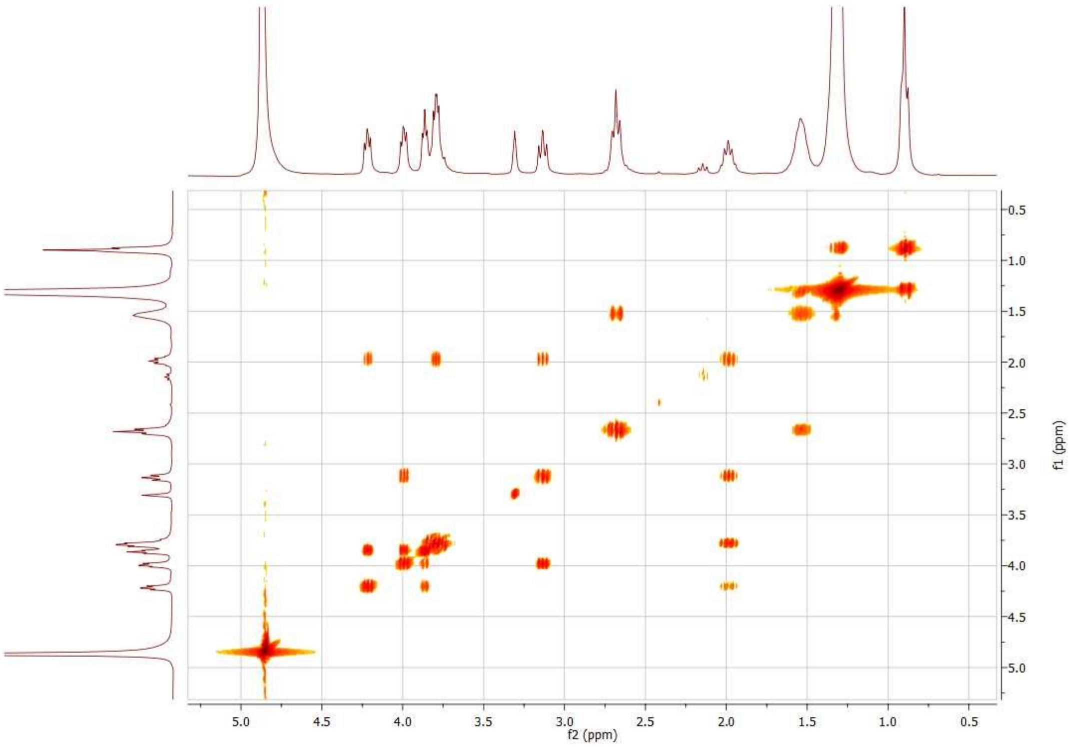

HSQC (CD_3OD): Compound **24**

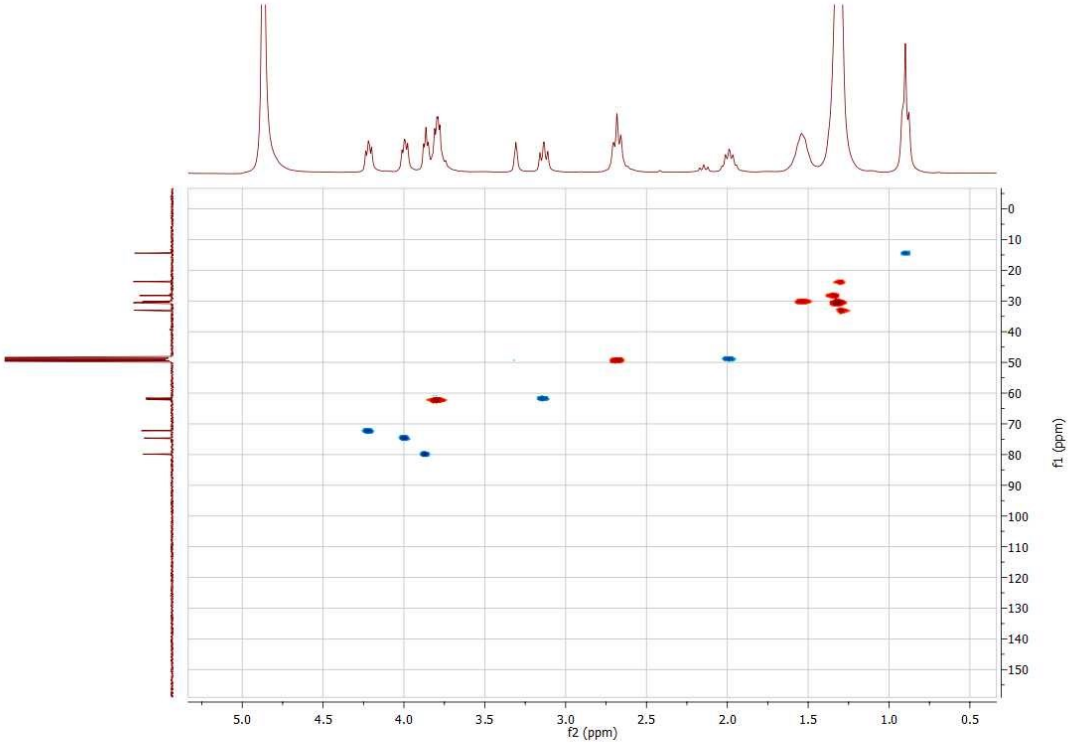

6-(((1*S*,2*S*,3*S*,4*S*,5*R*)-2,3,4-Trihydroxy-5-(hydroxymethyl)cyclopentyl)amino)hexanenitrile or 1-(6-Cyanohexyl)amino-α-D-*galacto*-cyclopentane (26)

^{1}H NMR (300 MHz, CD_3OD): Compound **25**

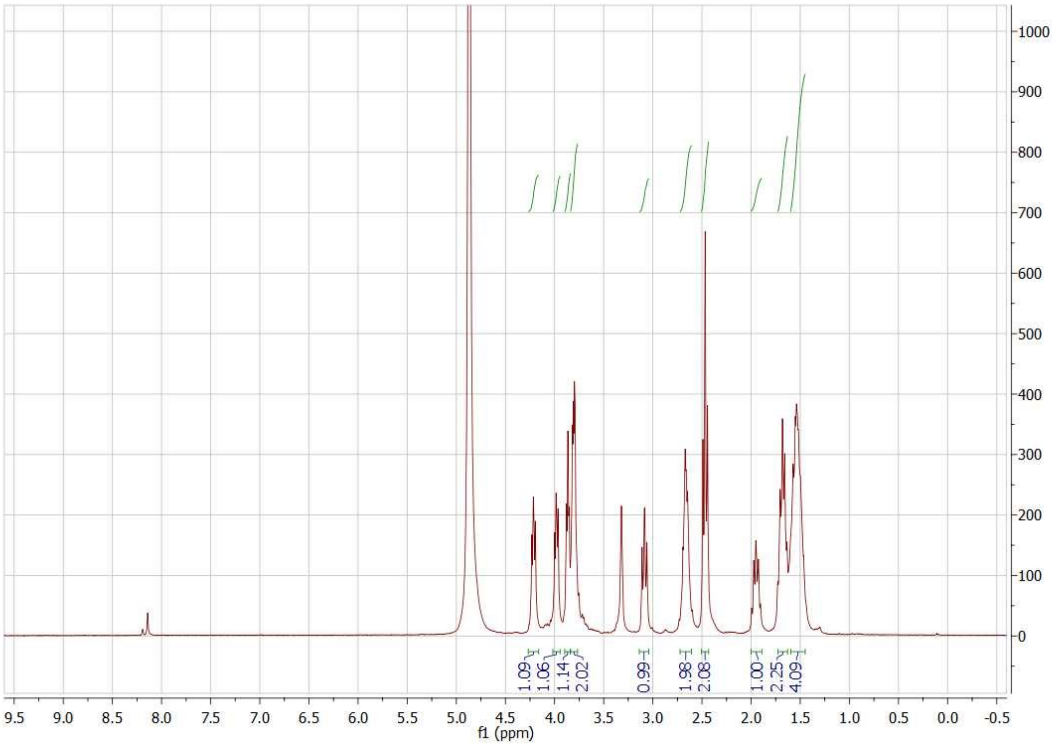

^{13}C NMR (75.5 MHz, CD_3OD): Compound **25**

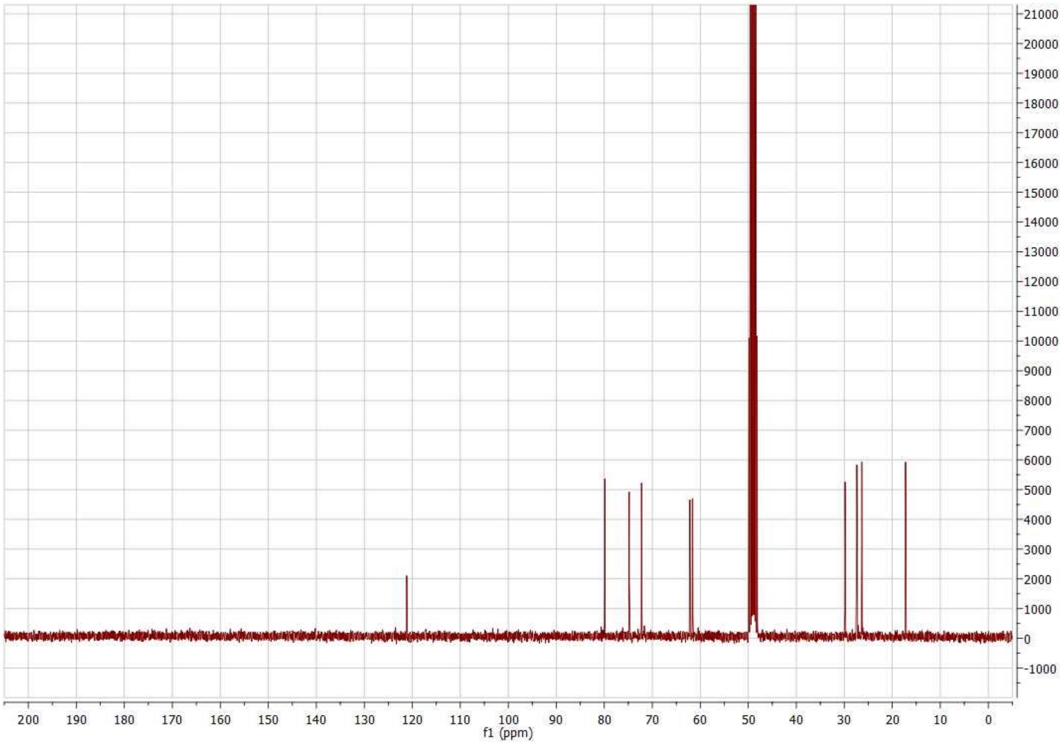

COSY (CD_3OD): Compound **25**

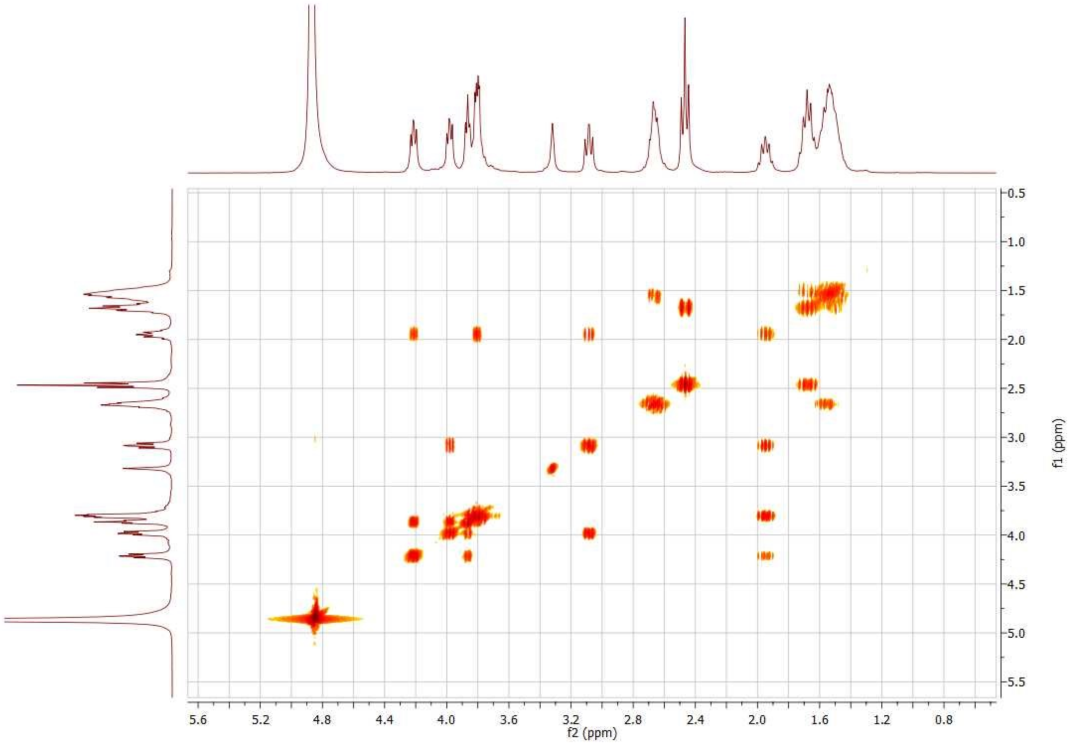

HSQC (CD_3OD): Compound **25**

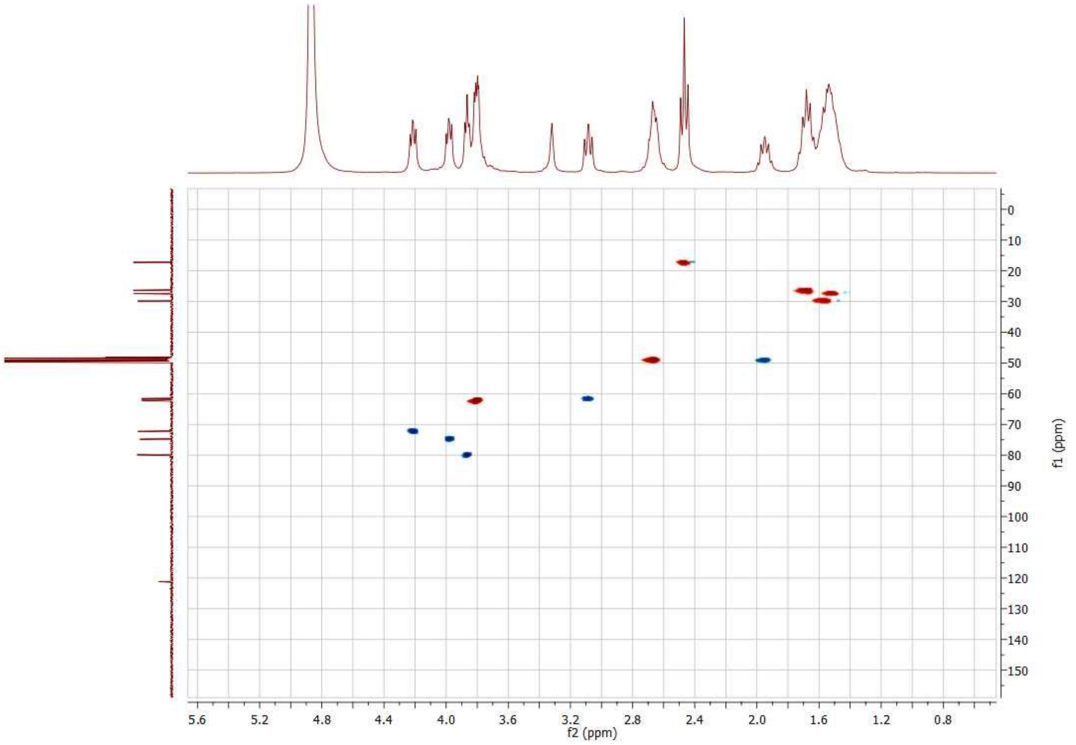

(1*S*,2*S*,3*S*,4*S*,5*R*)-4-[(6-Aminohexyl)amino]-5-(hydroxymethyl)cyclopentane-1,2,3-triol or **1-(6-Aminohexyl)amino-α-D-*galacto*-cyclopentane (xx)**

^{1}H NMR (300 MHz, CD_3OD): Compound **26**

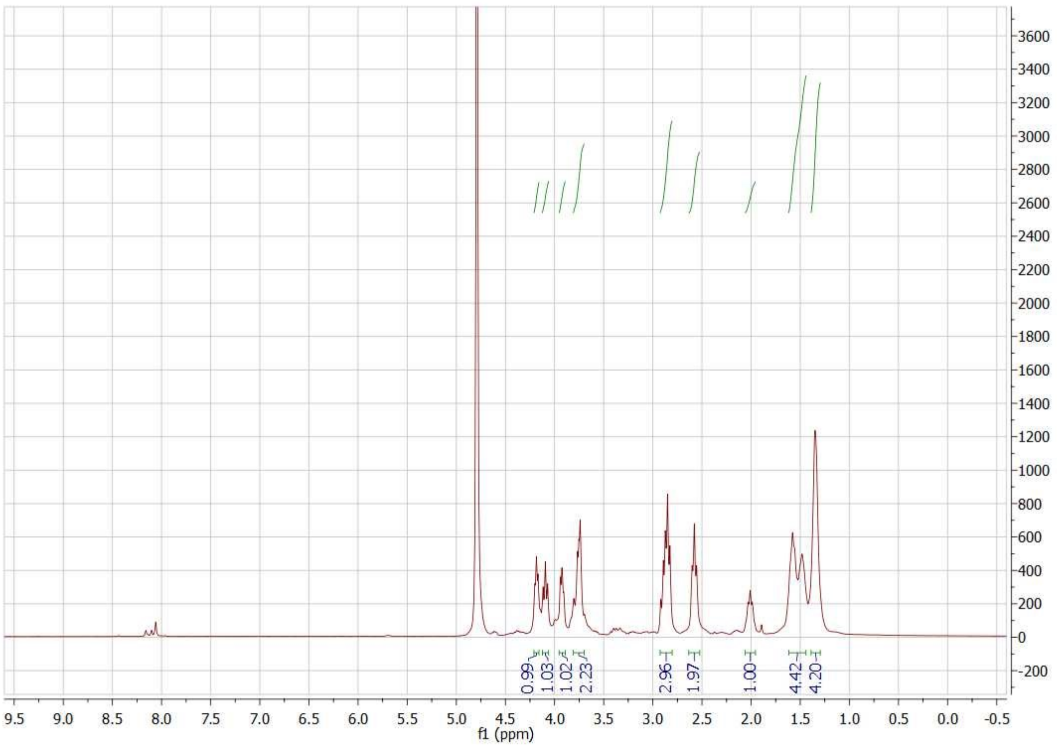

^{13}C NMR (75.5 MHz, CD_3OD): Compound **26**

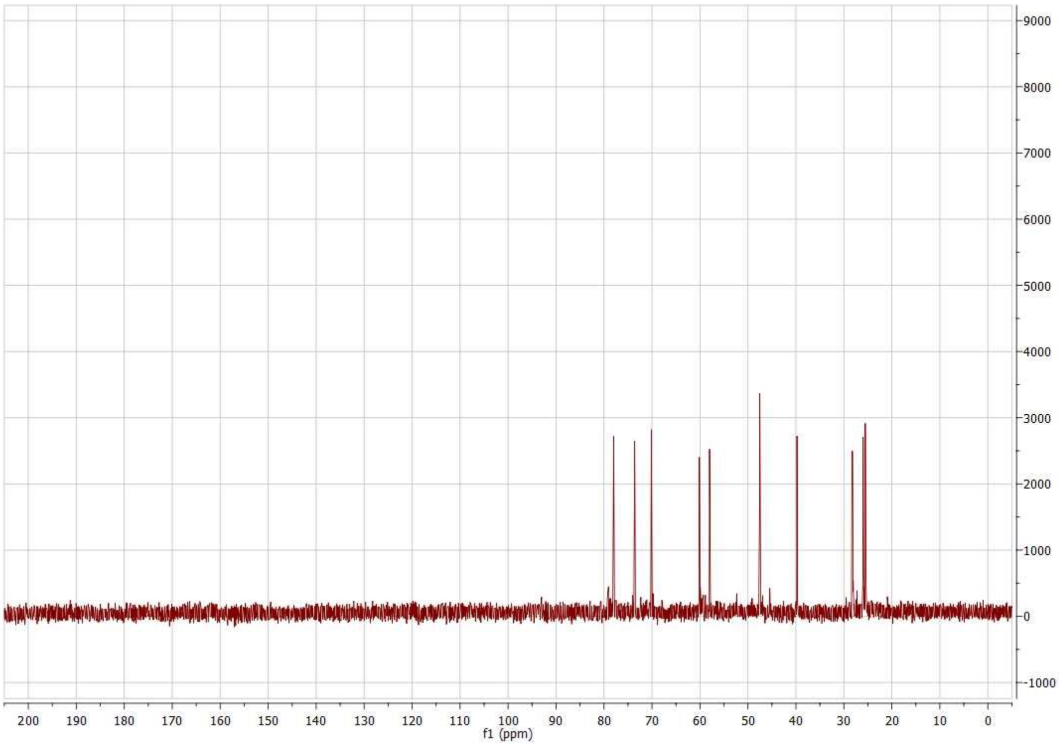

COSY (CD_3OD): Compound **26**

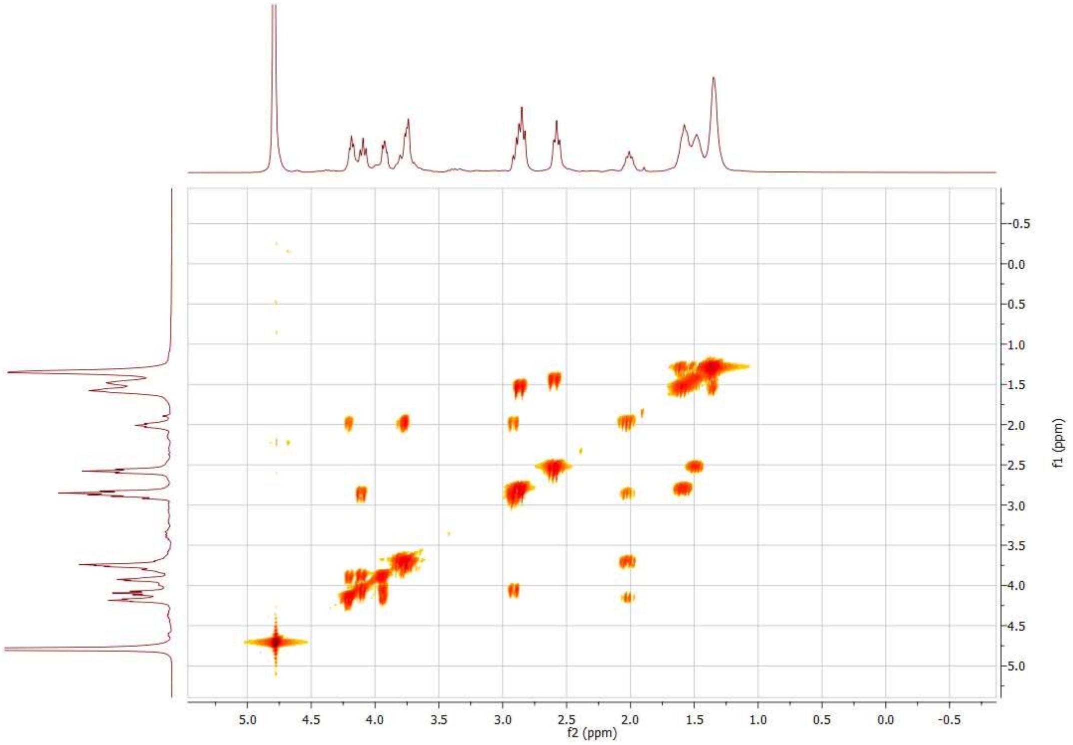

HSQC (CD_3OD): Compound **26**

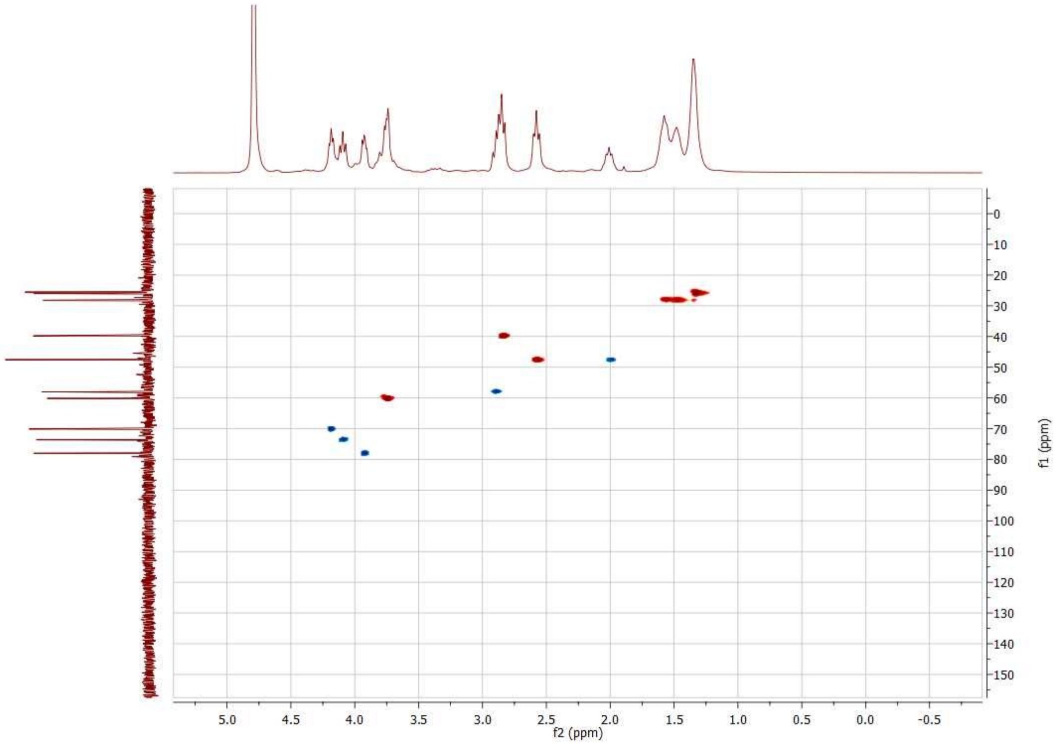

5-(Dimethylamino)-*N*-(6-{[(1*S*,2*S*,3*S*,4*S*,5*R*)-2,3,4-trihydroxy-5-(hydroxymethyl)cyclopentyl] amino}hexyl)naphthalene-1-sulfonamide or **1-(6-Dansylaminohexyl)amino-α-D-*galacto*-cyclopentane (27)**

^{1}H NMR (300 MHz, CD_3OD): Compound **27**

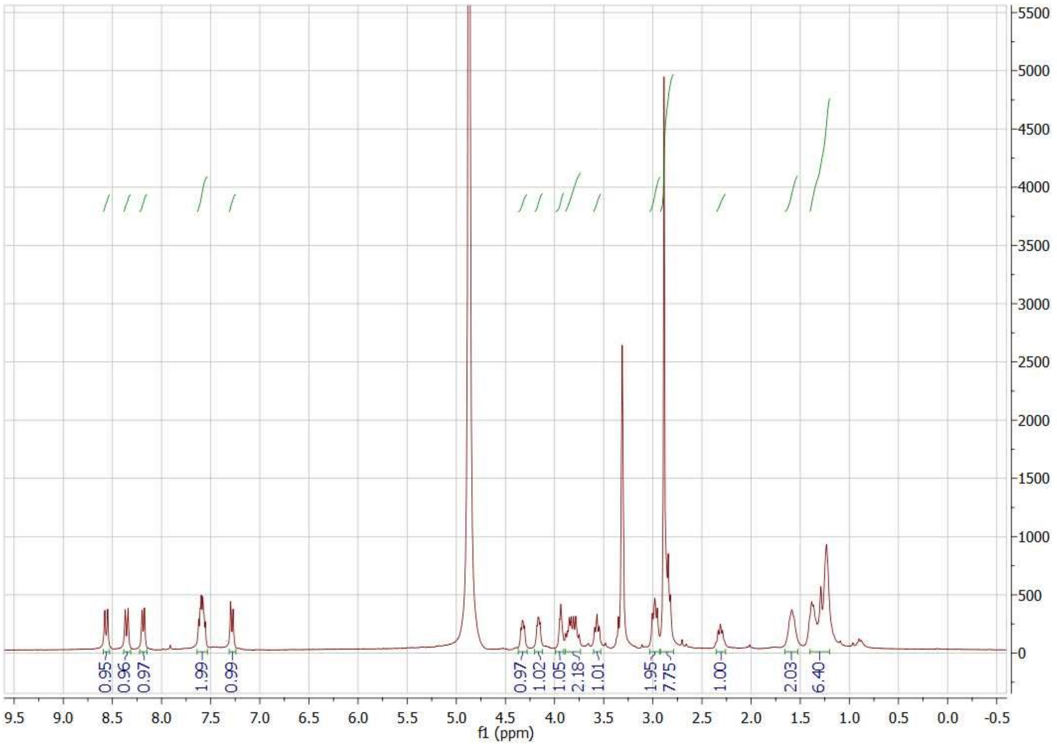

^{13}C NMR (75.5 MHz, CD_3OD): Compound **27**

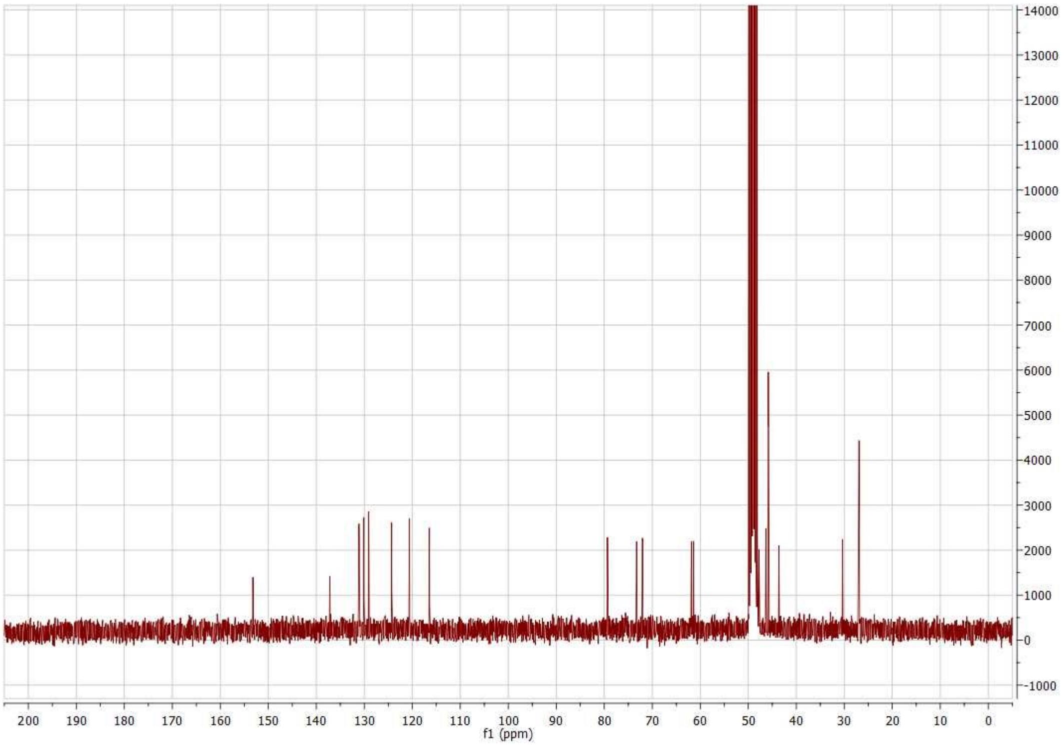

COSY (CD_3OD): Compound **27**

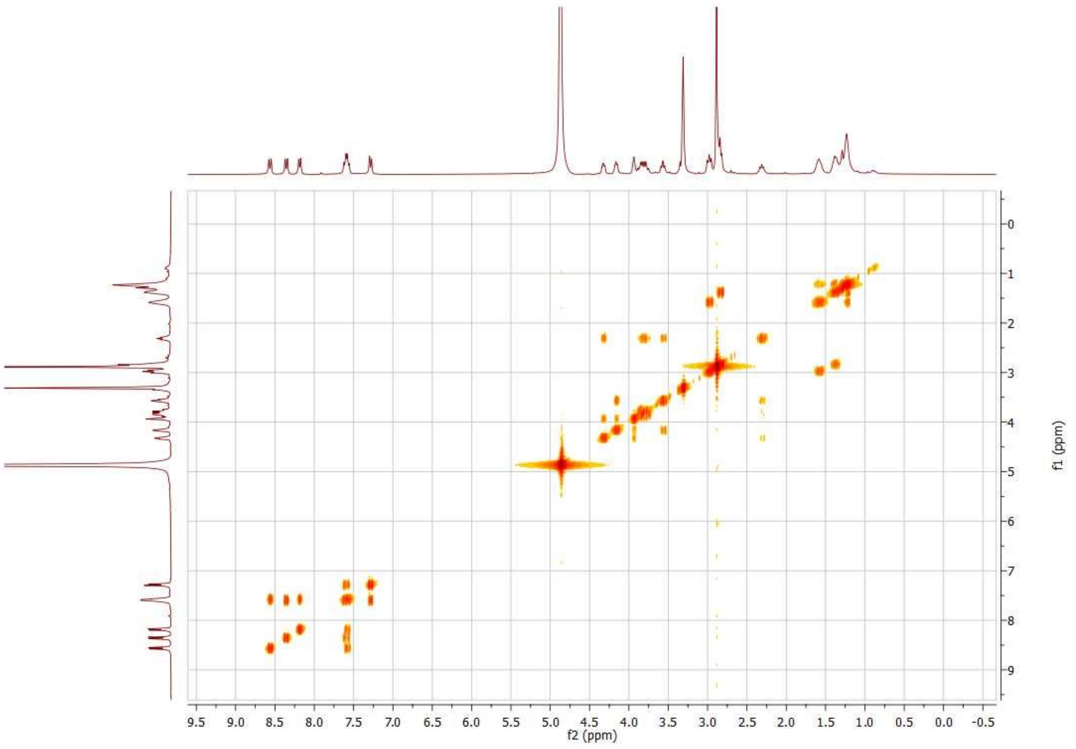

HSQC (CD_3OD): Compound **27**

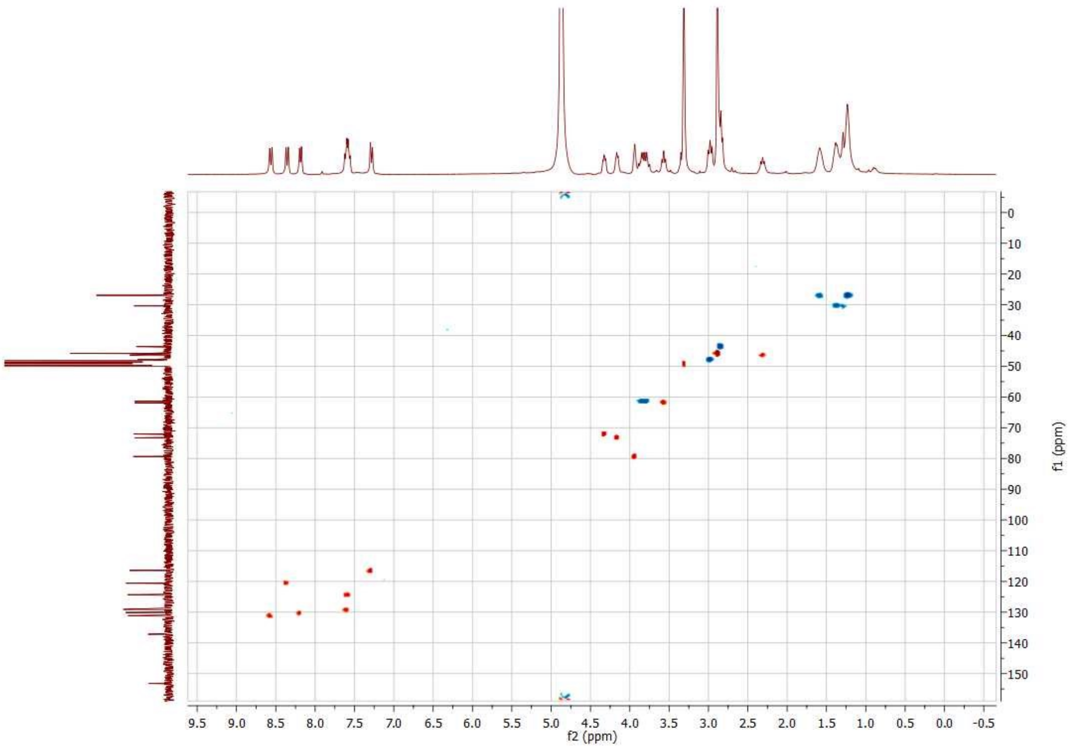

Supplement: RA-011-D1RA02507D-s001 [file RA-011-D1RA02507D-s001.pdf]
